# Machine learning for optical chemical multi-analyte imaging: Why we should dare and why it’s not without risks

**DOI:** 10.1007/s00216-023-04678-8

**Published:** 2023-04-18

**Authors:** Silvia E. Zieger, Klaus Koren

**Affiliations:** Aarhus University Centre for Water Technology (WATEC), Department of Biology, Section for Microbiology, Aarhus University, Ny Munkegade 114, 8000 Aarhus C, Denmark

**Keywords:** Supervised pattern recognition, XGBoost, Decision tree algorithm, Intensity-based sensing, pH, Dissolved oxygen

## Abstract

Simultaneous sensing of metabolic analytes such as pH and O_2_ is critical in complex and heterogeneous biological environments where analytes often are interrelated. However, measuring all target analytes at the same time and position is often challenging. A major challenge preventing further progress occurs when sensor signals cannot be directly correlated to analyte concentrations due to additional effects, overshadowing and complicating the actual correlations. In fields related to optical sensing, machine learning has already shown its potential to overcome these challenges by solving nested and multidimensional correlations. Hence, we want to apply machine learning models to fluorescence-based optical chemical sensors to facilitate simultaneous imaging of multiple analytes in 2D. We present a proof-of-concept approach for simultaneous imaging of pH and dissolved O_2_ using an optical chemical sensor, a hyperspectral camera for image acquisition, and a multi-layered machine learning model based on a decision tree algorithm (XGBoost) for data analysis. Our model predicts dissolved O_2_ and pH with a mean absolute error of < 4.50·10^−2^ and < 1.96·10^−1^, respectively, and a root mean square error of < 2.12·10^−1^ and < 4.42·10^−1^, respectively. Besides the model-building process, we discuss the potentials of machine learning for optical chemical sensing, especially regarding multi-analyte imaging, and highlight risks of bias that can arise in machine learning-based data analysis.

**Supplementary Information:**

The online version contains supplementary material available at 10.1007/s00216-023-04678-8.

## Introduction

Sensing multiple analytes at the same time and space has long been a key challenge in sensor development. Especially for biotechnological [1, 2], environmental [3-5], and medical [6] applications, where entangled biological processes lead to analyte transformations and the establishment of chemical gradients, multi-analyte sensors based on luminescent optical chemical sensors (the so-called optodes) have proven to be beneficial [7, 8] and are therefore in high demand. For instance, in heterogeneous systems such as biofilms, fragmented profiling of pH and O_2_ does not reflect the entire heterogeneous distribution within the biofilm, nor would monitoring with two individual sensors be able to capture the interdependence of these analytes and their combined influence on the biofilm [9, 10].

Hence, various approaches for luminescent-based optical chemical sensors are currently being investigated, all aiming at the simultaneous detection of multiple analytes at the exact same position with less complex and affordable equipment. The approaches span single indicators which show sensitivity to multiple analytes [11], to multi-layered systems that meet the spectral requirements of a given read-out system [12-16] (color camera with 3 to 4 channels), to the further development of existing read-out instrumentations [17]. However, despite recent progress in the field, certain limitations are inevitable. Specialized indicators normally require complex synthesis and are rarely commercially available. The combination of multiple indicators into a single sensor often leads to interactions between the respective indicators, such as energy transfer reactions, or to problems regarding the spectral separation of the respective overlapping emissions. Recently, we have shown that the later issue of overlapping emissions can be overcome by using hyperspectral imaging systems and spectral unmixing [17]. At the same time, we had to realize that while conventional methods in statistical data analysis are suitable for simple multi-analyte sensor systems where only the luminescence intensity of the indicators changes as a function of analyte concentration [17], these methods fail when indicators also undergo a spectral shift at the same time. In this case, the interactions and dependencies of the indicators become too complex. Analysts are therefore no longer able to deduce an unambiguous and universal model that considers all potential cross-sensitivities. To overcome this and decipher complex and nested datasets, machine learning algorithms (ML) offer great potential. ML exploits the ability of computers to learn from (training) data, recognize patterns in nested datasets, and automate the construction of analytical models. Since their emergence in the second half of the twentieth century, ML models have been applied in a variety of fields, including life and environmental sciences for predicting extreme natural events using remote sensing [18], enabling smart sensor systems [19], and drug delivery [20, 21]. Some interesting work using ML approaches has already been done related to optical sensors [22, 23]. Expanding on this work, we now want to apply ML models to enable multi-analyte imaging in 2D to visualize the heterogeneity of biological environments and the distributions of multiple analytes in 2 dimensions simultaneously. While other sensing approaches, especially fiber-based single-point sensor approaches might face an operational challenge of creating large (training) datasets, which is, however, a prerequisite for training ML models to derive an underlying trend according to the large number theorem [24, 25], it is the inherent nature of imaging to record hundreds of quality sample data within one single image acquisition.

Therefore, we present a novel proof-of-concept approach for optical chemical multi-analyte imaging using a machine learning (ML) model. Using a dual analyte sensor for pH and dissolved oxygen, we demonstrate the potential of ML for nested and intercoupled emission spectra of optical chemical sensors. Using a hyperspectral camera as the read-out system provides us with a sufficiently large amount of data within one single image acquisition, where each image pixel contains high-quality information over the entire spectral range between 470 and 900 nm. In the following, we first introduce the problem of a complex and nested dataset for the dual analyte sensor, which cannot be solved with conventional statistical models. We then describe the ML model as well as its performance and conclude with a discussion about the benefits and risks of the novel approach for optical chemical multi-analyte sensors.

### Material and methods

Refer to the supplementary materials for more information on algorithm optimization, validation of the final ML model, or its visualization. In addition, examples of the raw calibration data for the 2-layered optical chemical sensor as well as a spreadsheet containing the prepared calibration data can be downloaded from the Mendeley data repository [26]. Due to the available space at the repository, we are only able to share examples of the original hyperspectral fluorescence images.

### Materials

The O_2_-sensitive indicator dye platinum(II)-meso-tetraphenyl-tetrabenzoporphyrin (Pt-TPTBP) and the reference dye Macrolex Fluorescence Yellow (MFY 10GN) were purchased from Frontier Scientific (frontiersci.com, Logan; USA) and Lanxess AG (lanxess.com, Köln; Germany), respectively. The lipophilic pH indicator HPTS (1-hydroxypyrene-3,6,8-tris-bis(2-ethylhexyl)sulfonamide was provided by Dr. Sergey Borisov, Graz University of Technology, Austria) [27]. Additional chemicals for sensor fabrication and calibration, such as polystyrene (PS. MW 192.000 g·mol^−1^), polyurethane-based hydrogel (HydroMed D4), sodium sulfite (Na_2_SO_3_), ethanol, and toluene, were bought from Sigma Aldrich (sigmaaldrich.com, St Louis; USA), Advan Source biomaterials (advbiomaterials.com, MA; USA), and Merck KGaA (merckgroup.com, Darmstadt; Germany). The monocrystalline diamond powder was purchased from Pureon (pureon.com, Lengwil; Switzerland). All buffer materials (sodium phosphate monobasic monohydrate NaH_2_PO_4_ · H_2_O and dihydrate NaH_2_PO_4_ · 2H_2_O) were obtained from Sigma Aldrich (sigmaaldrich.com, St Louis; USA). The PET support foil (Lumirror 4001, 125 µm) was obtained from Puetz Folien (puetz-folien.com, Taunusstein; Germany). All chemicals were used as received.

### Optode fabrication

A sensor cocktail was prepared for the fabrication of the optodes according to literature [28]. First, the O_2_-sensitive layer was prepared, for which 0.94 mg of the Pt-TPTBP indicator and 0.86 mg of the MFYreference dye were dissolved in 1 g of a 10%w/w polymer matrix of PS (in toluene). The sensor cocktail was knife-coated onto a dust-free PET support foil using a film applicator (Byk-Gardner GmbH, Germany) yielding a ~ 12-µm-thick sensor layer after solvent evaporation. For the pH-sensitive layer, 0.95 mg of the lipophilic HPTS and 48 mg monocrystalline diamond powder, serving as a signal enhancer, were dissolved in 1 g of a 10%w/w solution of D4 (in ethanol:water, 9:1 w/w). This sensor cocktail was knife-coated onto the top of the well-dried O_2_-sensitive layer yielding a ~ 10-µm-thick pH layer after solvent evaporation. The total thickness of the dual analyte optode was thus ~ 22 µm. In addition to the dual analyte sensor, single sensors consisting of only one layer, sensitive to either pH or O_2_, were also coated with the same cocktail compositions as described previously.

### Imaging setup and optode calibration

**Fig. 1:**
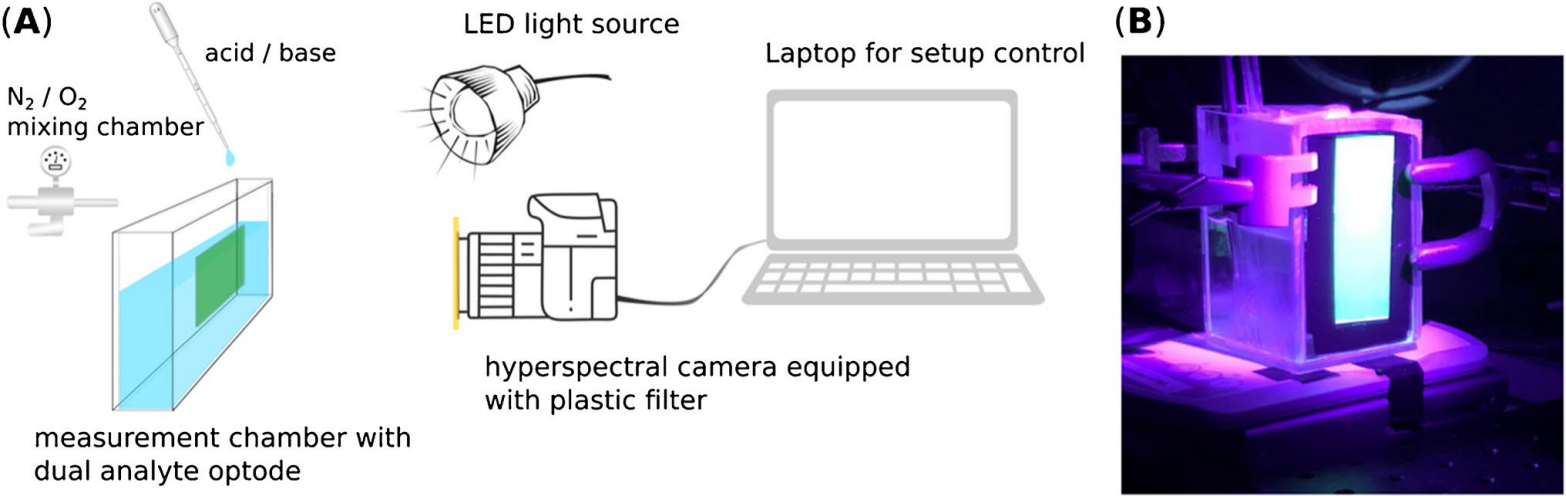
Schematic representation of the measurement setup used for measuring and calibrating the optical chemical dual-sensor for pH and dissolved O_2_ (**A**) and real image of the fluorescence of the dual analyte optode upon excitation with a high-power LED (**B**)

The setup was built similarly to that described in a previous paper of ours with some adaptations to suit the current dual analyte sensor [17]. In Fig. 1, a schematic of the imaging setup is shown for clarification. In short, the setup consisted of a hyperspectral camera (imec SnapScanVNIR camera; imec-int.com, Belgium) equipped with a color-corrected objective (Apo-Xenoplan lens, f2.0; Schneider-Kreuznach GmbH, German). A plastic filter (#10 medium yellow; LEEfilters.com, UK) was placed in front of the objective to reduce background fluorescence. The camera was connected to a PC and controlled using the manufacturers’ hyperspectral image-recording software (HSI Snapscan v1.4.1.0; imec-int.com, Belgium). For image acquisition, the camera was set to scan the full image frame (1088 × 2048 pixels) and the full spectral wavelength range (470–900 nm) with a pixel step of 3 nm and an integration time of 5 ms. The pixel blur and binning were set to 0 and 1, respectively. The dual analyte sensor foil was excited with a high-power LED light source (460 nm; LED Hub, Omicron Laserage Laserprodukte GmbH, Rodgau, Germany) equipped with a 1-m liquid light guide and a collimating lens. The LED light source was controlled via a PC running the manufacturers’ software. The dual analyte sensor foil of approximately 2.5 × 8 cm^2^ was taped on the inner transparent glass wall of a buffer-filled measurement chamber. Excitation and imaging of the sensor foil were done frontally through the chamber wall.

Calibration of the dual analyte optode was performed similarly to as it is described in the literature; however, some adjustments were made to suit the dual analyte optode [14, 28]. For pH calibration, the pH of the phosphate buffer (0.1 mol·L^−1^ with an ionic strength of 0.377 mol·L^−1^) was adjusted by using 1 mol·L^−1^ HCl and NaOH solutions. Oxygen levels were altered by using compressed O_2_ and N_2_ (Air Liquide S.A., airliquide.dk; Taastrup, Denmark), which were mixed with a gas mixer (Red-y-compact; Vögtlin Instruments GmbH; Muttenz, Switzerland). At the lowest O_2_ calibration points, sodium sulfite was added as an extra O_2_ scavenger to ensure fully anoxic conditions. All measurements were performed at the same constant temperature (22.5 ± 0.5 °C). A pH meter (PHM210 Meterlab, Radiometer Analytical, Lyon, France) facilitated the monitoring of the pH throughout the calibration. O_2_ levels and temperature were monitored with a fiber-optic O_2_ phase-fluorimeter (FireSting GO_2_; PyroScience GmbH, Aachen, Germany) equipped with a robust O_2_ sensor (OXROB3; PyroScience GmbH, Aachen, Germany).

### Spectral characterization of individual layers of the dual analyte optode

For full spectral characterization of the single and dual analyte optodes, additional fluorescence and excitation spectra were acquired with a ClarioStar Plus plate reader (BMG Labtech, Ortenberg, Germany) at room temperature and different pH and oxygen conditions. The optodes were taped into a 12-well plate and filled with 1 mL of phosphate buffer, and the pH was adjusted by using 1 M HCl and NaOH solutions. The O_2_ levels were either reached by shaking the buffer solution before filling it into the well or by adding a few drops of a 2% solution of sodium sulfite. For the excitation spectra, the excitation wavelength was scanned between 350 and 700 nm (slit width 10 nm, increment 2 nm), while the emission wavelength was set to 770 nm (slit width 10 nm). To record the fluorescence spectrum, the emission wavelength was scanned between 420 and 840 nm (slit width 10 nm, increment 2 nm), and the excitation wavelength was set to 380 nm (slit width 10 nm).

### Image analysis and data processing

#### Required programming packages

The radiometric correction of the raw hyperspectral image is done using a MATLAB script that can be obtained from the camera manufacturer upon request (hsisupport@imec.be). The image analysis, data processing, and the ML model were coded in Python 3.7.4 (python.org) using the following Python packages: for loading and processing hyperspectral images, we used SpectralPython (SPy, spectralpython.net), matplotlib (matplotlib.org), and Python Imaging Library (PIL; pypi.org/project/Pillow); for spectral fitting and solving the integration and the optimization problem, SciPy (scipy.org) and the nonlinear least-square fitting (lmfit; lmfit.githu-b.io) were used. Further packages required are NumPy, pandas, math, random, time, glob, pathlib, os, h5py, and andxlrd. All libraries required were to date at the time the paper was submitted. The Python code can be downloaded from GitHub (github.com/silviaelisabeth/ML_for_pHandO2) and is openly accessible.

#### Performance analysis

While classification models in ML can be assessed and evaluated straightforwardly based on certain performance measures such as their accuracy, this is not the case for regression models. In regression, the model performance is reported as its deviation or error from the expected target values. While there are various approaches to assessing the regression performance of a model, the commonly used error metrics are the root mean square error (RMSE) and the mean absolute error (MAE) [29, 30].

##### Root mean square error (RMSE)

The root mean square error is also called root mean square dispersion and measures the difference between the estimated (*y*_*i*_) and the expected target (*x*_*i*_) values. The difference between these values is first squared and then averaged across the entire data samples. Finally, the square root is calculated. The RMSE determines the average magnitude of the error and is a negatively oriented scoring rule, i.e., the lower the error, the better the model prediction performance. However, RMSE is less robust towards outliers:

1 $$RMSE=\sqrt{\frac{{{\sum }_{i=1}^{N}\left({y}_{i}-{x}_{i}\right)}^{2}}{N}}$$

with *y*_*i*_ being the estimated value and *x*_*i*_ being the expected value for the *i*th sample. *N* is the number of samples in the given dataset.

##### Mean absolute error (MAE)

The mean absolute error instead does not take the square of the difference between observed and predicted values but the absolute value. It is thus more robust towards outliers and does not penalize larger errors more than smaller ones:

2 $$MAE= \frac{{\sum }_{i=1}^{N}\left|{y}_{i}-{x}_{i}\right|}{N}$$

with *y*_*i*_ being the estimated value and *x*_*i*_ being the expected value for the *i*th sample.* N* is the number of samples in the given dataset.

### Results and discussion

In optical chemical sensing, where changing spectral properties of an analyte-sensitive indicator are correlated with the analyte concentration, the situation can quickly become complex. Not only effects such as leaching or bleaching may alter the sensor over time, but also due to the inhered interaction of individual components within the sensor with each other through energy or electron transfer reactions. This makes the evaluation of luminescence spectra more complex since these alternations and cross-interferences must be considered when calibrating the indicators, especially if those cross-interferences do not remain constant [31].

**Fig. 2:**
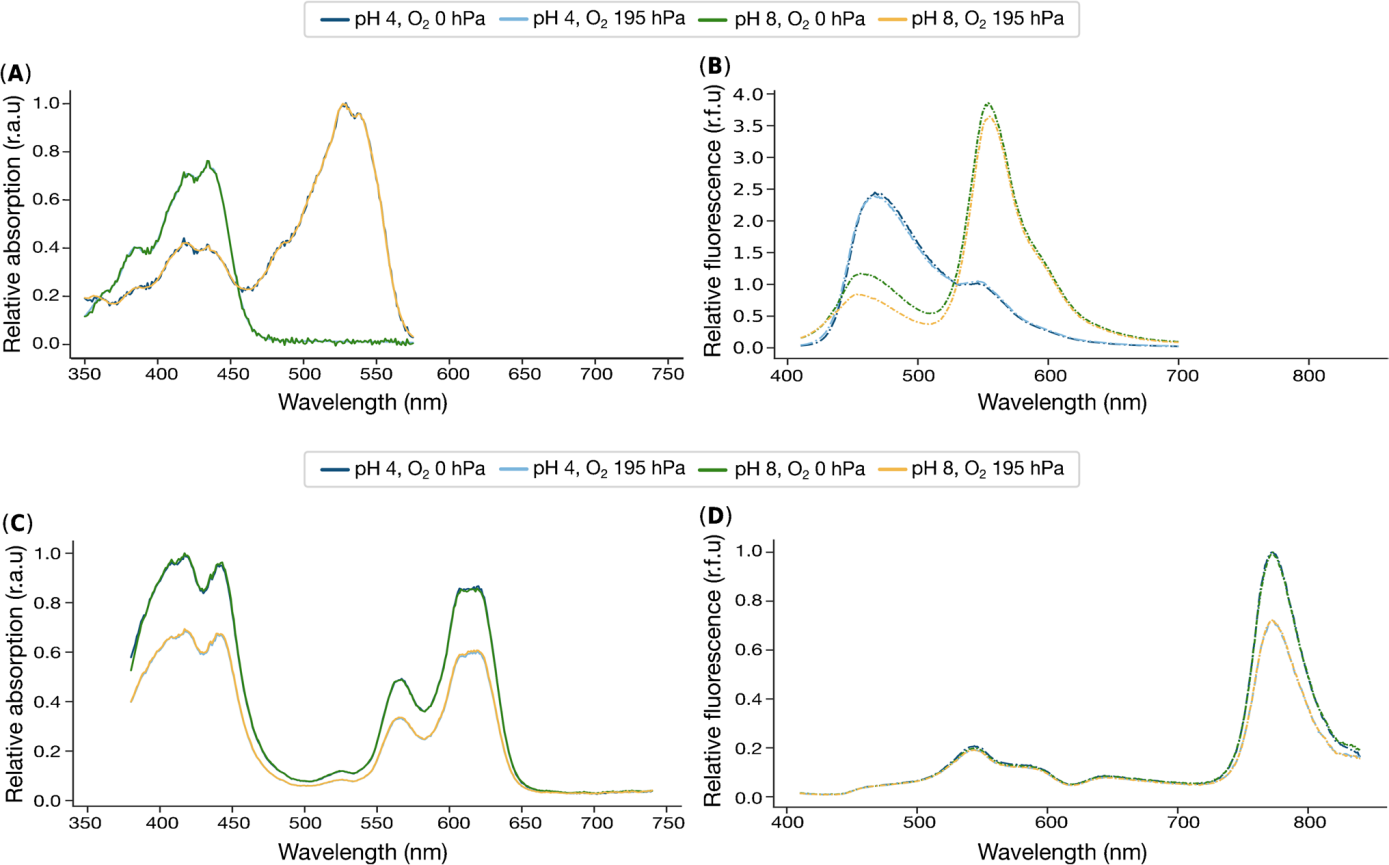
Spectral characterization of the single optode layers recorded on the ClarioStar Plus plate reader under different pH and O_2_ conditions. The excitation spectra of **A** lipophilic HPTS as a pH-sensitive dye and **C** Pt-TPTBP as an O_2_-sensitive dye are shown in the left panels, while the emission spectra of **B** the pH indicator and **D** the O_2_ indicator are shown in the right panel. Note that the O_2_-sensitive sensor layer also contains macrolex fluorescent yellow as the reference dye. While in **A**, **C**, and **D**, the fluorescence intensity is displayed relative to the maximum intensity, in **B**, the intensity is displayed relative to the isosbestic point at 530 nm

Figure 2 displays such a complex situation for the simultaneous imaging of pH and dissolved O_2_. The figure depicts the spectral excitation/emission characteristics of the individual layers of the optical chemical dual analyte sensor for pH (Fig. 2A–B) or dissolved O_2_ (Fig. 2C–D). While the two-layered structure of the optode should prevent close-proximity energy transfer reactions, such as Förster resonance energy transfer (FRET) or photoinduced electron transfer (PET), reabsorption of luminescence can still occur when the excitation and emission spectra of the indicators involved overlap [32].

Figure 2A and D reveal that reabsorption may occur to some extent, particularly under basic conditions, as the reference indicator, macrolex fluorescence yellow emits between 500 and 600 nm (Fig. 2D), which overlaps with the absorption of lipophilic HPTS (yellow and green curve in Fig. 2A). However, the reabsorption of the O_2_ sensor layer (combination of macrolex fluorescence yellow dye and oxygen-sensitive Pt-TPTBP dye) (Fig. 2C) is predominant due to the overlapping excitation of the lipophilic HPTS dye (Fig. 2B). In particular under acidic conditions, when the pH indicator emits between 400 and 550 nm, reabsorption by the O_2_ sensor layer (Fig. 2C) can occur. However, at higher pH values, this resonance and reabsorption are reduced since the overlap between emission and absorption is less. The complex and nested combination of several different effects creates a situation that cannot be predicted and accounted for in one or a few polynomial functions as it is required by conventional approaches to signal deconvolution.

**Fig. 3:**
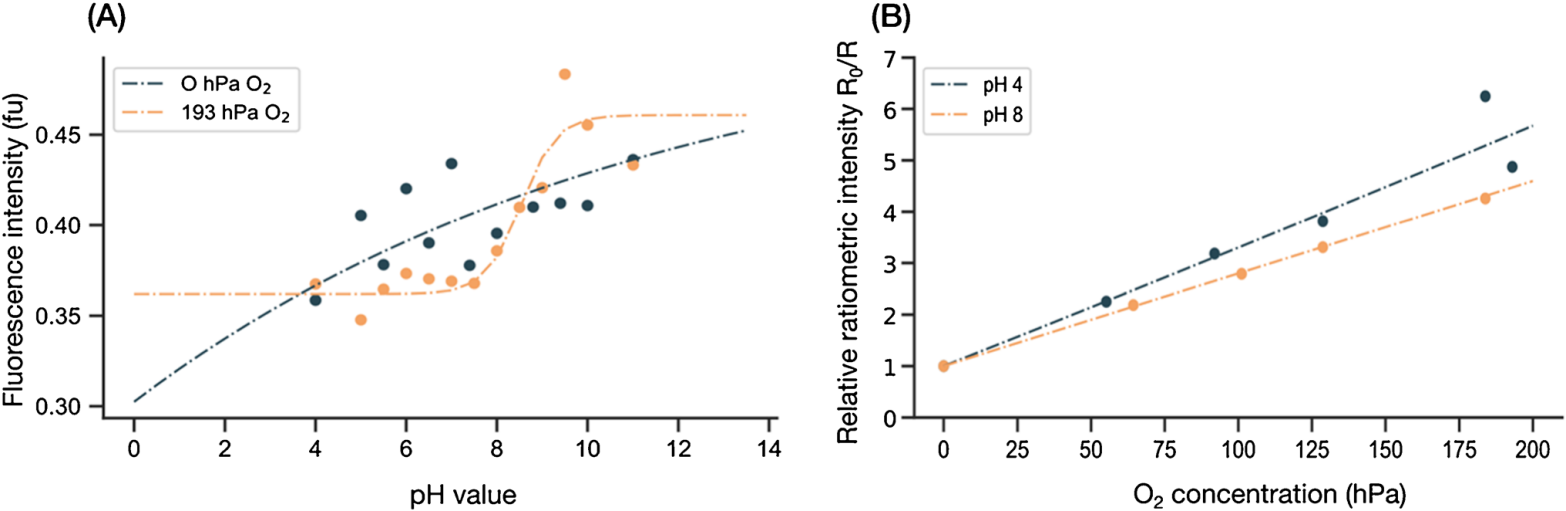
pH and O_2_ calibration of the dual analyte optode under constant condition of the respective other analyte. **A** pH calibration between pH 4 and 11 under anoxic (0 hPa) and air-saturated (195 hPa) conditions. **B** O_2_ calibration is displayed as ratiometric intensity relative to the reference indicator, macrolex fluorescence yellow, while the pH is kept constant at either pH 4 or pH 8. The dashed curves in both panels represent the hypothetical calibration curves of the analytes if the respective standard calibration functions for the individual analytes, i.e., Boltzmann fit for pH calibration and simplified Stern–Volmer fit for O_2_ calibration, were applied to the calibration points

Figure 3 subsequently illustrates this nested and intercoupled situation with spectral cross-interferences when it comes to calibrating the different analytes. While Fig. 3A displays pH calibration data of the dual analyte optodes at two different O_2_ concentrations (anoxic and air-saturated), Fig. 3B displays O_2_ calibration data at two different pH values (4 and 8). The dashed curves in the panels represent hypothetical calibration curves if respective standard calibration fit functions were applied to the calibration data to calibrate the individual analytes. As can be seen from the graphs and especially from Fig. 3A, the fitted calibration curves fail to describe the calibration data well as there is cross-dependence in both calibrations. While most of the calibration data might be solved individually by conventional fit functions, the pH calibration under anoxic conditions as well as the interpolation of all other analyte combinations can hardly be solved by applying conventional analysis methods and calibration functions. However, it is important to note that the indicators chosen in this example show a spectral overlap and were specifically chosen to also demonstrate the limitations in selecting commonly used (available) indicators. By combining other indicators with less spectral overlap, this issue could be eliminated or reduced; this often requires the synthesis of specialized indicators, which for various reasons is not always possible.

That is the point ML modeling comes into play as an alternative approach. The advantage of ML models lies in their capability of finding underlying patterns and parameter correlations in nested and interconnected datasets whose complexity and dimensionality are beyond human imagination. For the modeling, we decided to use the absolute fluorescence response of the dual analyte optode, as opposed to the usual approach in optical–chemical sensing, which uses ratiometric intensity relative to the reference indicator. Our decision was based on the fact that, in our tests, the former approach yielded slightly better results than the latter one.

**Scheme 1:**
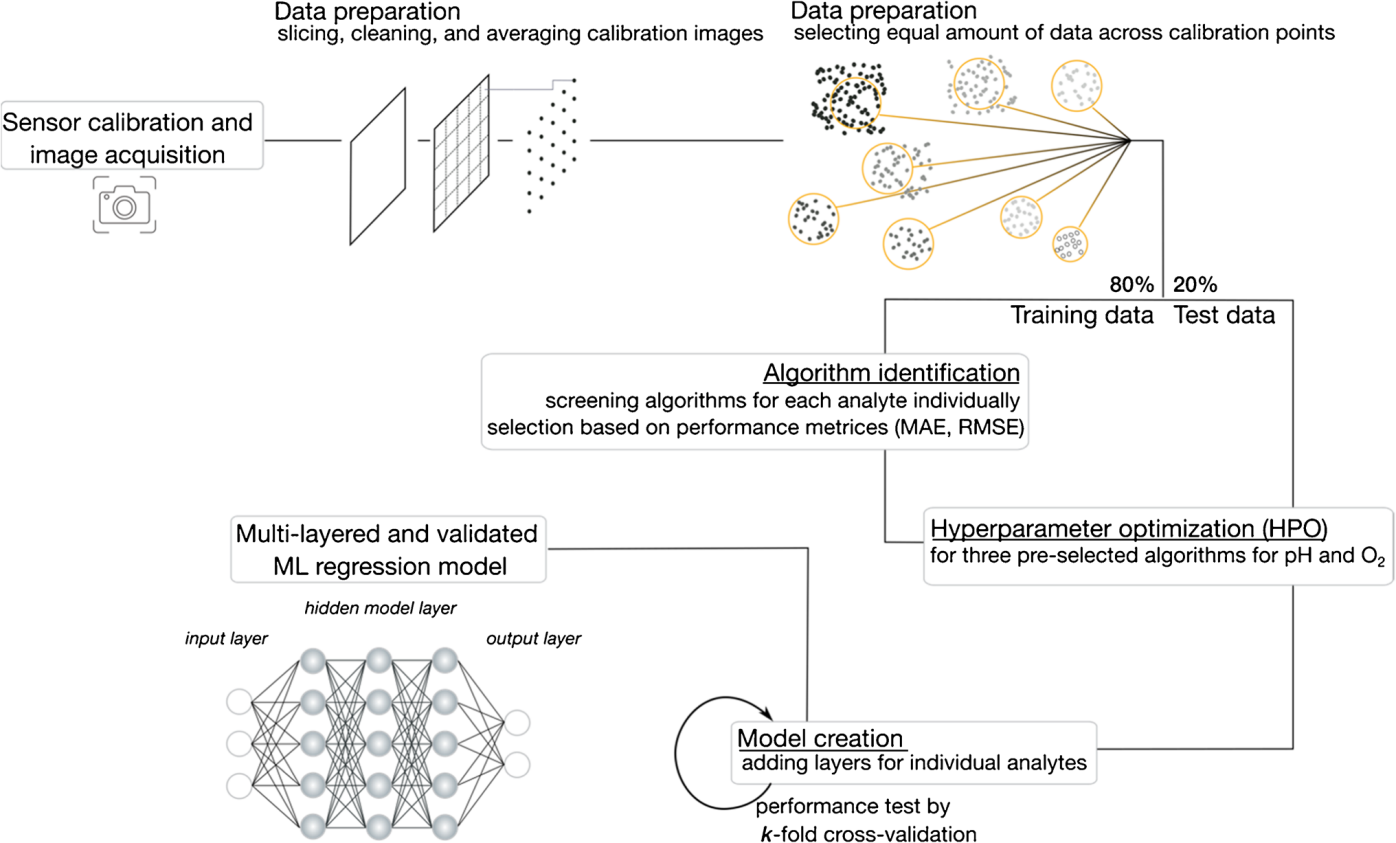
Overview of the workflow conducted to build up the multi-layered machine learning model for simultaneous detection of pH and dissolved O_2_

In the following, we first explain data extraction and preparation, which are a crucial step in modeling, and describe important aspects that can affect model performance. We then describe the process of model building and optimization, followed by a description and validation of the final model for simultaneous imaging of pH and dissolved O_2_. Especially during the validation step, the advantage of ML modeling becomes clear, but at each step, we emphasize the risks of bias that can impact the overall model performance. Scheme 1 provides an overview of the workflow with all processes conducted from initial data acquisition to the final machine learning model.

### Data preparation

The first key step in building a strong ML model is to provide a suitable dataset on which the algorithm can train and deduce an underlying (hidden) pattern. For a well-performing ML model, a suitable dataset means providing a large or even big set of samples that contain balanced and high-quality information to follow the large numbers theorem [24, 25, 33]. Although it depends on the individual problem and its complexity, computer scientists argue that a rule of thumb is at least 1000 samples for a suitable dataset. In some cases, when the amount of data is not a limiting factor, researchers may apply dimension reduction techniques such as principal component analysis, factor analysis, or linear discriminant analysis to enhance information density and remove unwanted noise from random variables before applying further regression algorithms [34, 35]. However, applying dimensional reduction techniques may also filter out relevant information for subsequent regression algorithms to find the underlying patterns. Therefore, we opted for an outlier removal test to ensure data quality instead of a dimension reduction technique. In addition, our preliminary tests (not shown here) demonstrated that this approach led to better results without sacrificing relevant information.

Hence, to match the first requirement and extract a sufficiently large amount of spectral data from the optode image, we selected a homogeneous region of interest (RoI) from the optode calibration image. However, unlike the usual approach in optical chemical imaging, we did not average over a larger area of the optode but chose smaller sections of 5 × 5 pixels for the RoIs, cleaned the data from outliers with an interquartile range, and calculated the median average of each RoI. In this way, we obtained 7196 oxygen samples and 6476 pH samples while mitigating the noise of the optode images. A table has been compiled from these processed data, and the interested reader may download the calibration data from the publicly accessible repository Mendeley data [26].

**Fig. 4:**
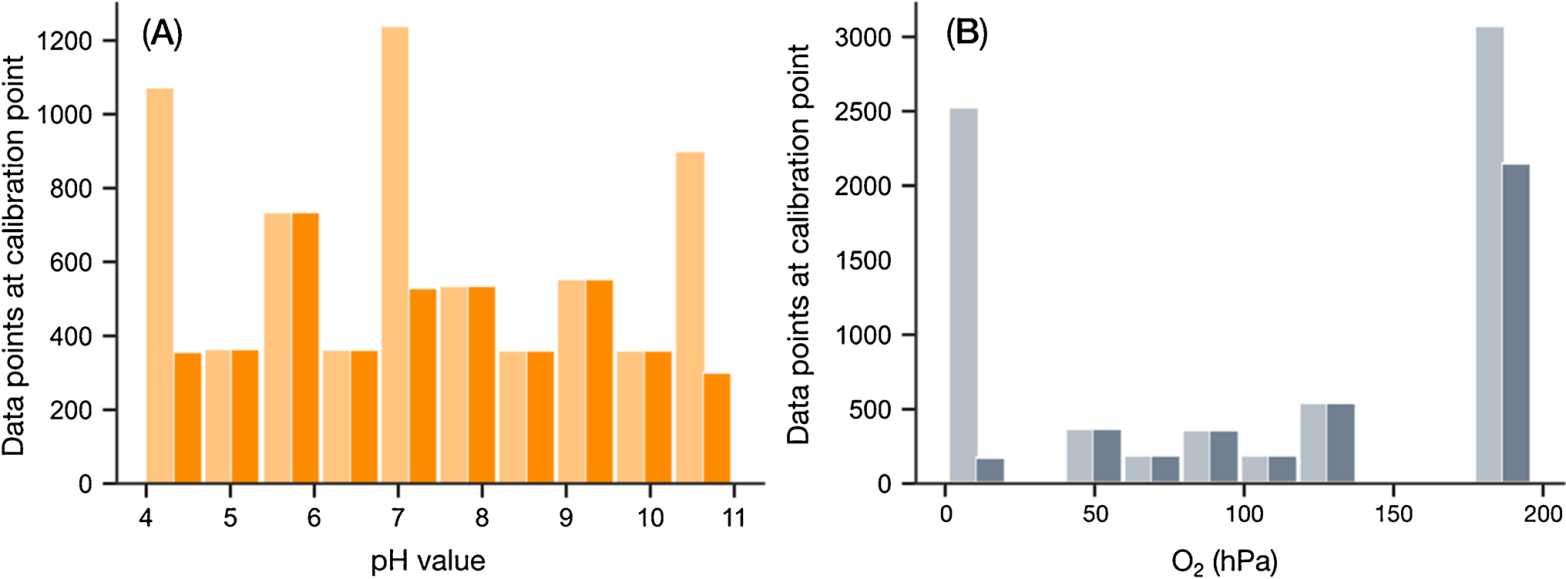
Dataset adjustment of the unbalanced calibration dataset by reducing the number of data points used where data are prevailing. The amount of data points used is adjusted to the general median. The adjustment is performed separately for each analyte. For each panel, the original distribution of the dataset is shown in light color, while the more balanced dataset is shown in dark colors, i.e., (**A**) in orange for pH and (**B**) in gray for O_2_

In the next step, we examined the distribution of sample points across calibration points to tailor the dataset to be balanced, i.e., each calibration point is almost equally represented in the dataset. This is critical to avoid bias in model accuracy and to prevent the model from being trained on a hidden bias that may stem from artifacts but has nothing to do with the actual feature correlations (overfitting). As can be seen in Fig. 4, the distribution of the sample data we obtained for each calibration point (original dataset shown in bright colors) is highly imbalanced notably for the oxygen calibration where calibrations at air saturation and under anoxic conditions are prevailing. Thus, we reduced the prevailing samples by averaging larger groups of pixels and ultimately obtained sample sizes of 2506 samples for oxygen and 4450 samples for pH, respectively. The distribution of the final dataset is shown in dark colors in Fig. 4.

### Machine learning regression model

When screening the literature for an appropriate machine learning algorithm, one comes across a great variety of machine learning algorithms applied to a wide range of topics and problems, including research questions in life and environmental sciences [1, 2]. The field is constantly evolving, with new algorithms being introduced to solve increasingly complex problems in less time. Each of them with different strengths and potentials, but not all of them are applicable to every research question or sometimes even unnecessarily complex in terms of computational power or do not match the given data or problem at hand. Interested readers can read more about other ML models in the referenced publications [29, 33–37]. Subsequently, we describe how we selected and optimized an appropriate ML model based on the given dataset and validated its ultimate performance.

#### Model identification

The measured calibration dataset is best described as a structured dataset summarizing the spectral responses of the dual analyte sensor along the entire wavelength between 470 and 900 nm at different pH and O_2_ conditions. In addition to the spectral response of the dual analyte sensor (the so-called features of the dataset), the specific pH and O_2_ concentrations during calibration are known. Thus, the calibration dataset can be described as a labeled, structured dataset with an additional target vector that allows the use of supervised ML algorithms. Another important point is that although the structured dataset provides discrete calibration points, it must be possible to obtain continuous results in subsequent measurements, which hence requires a regression model rather than a classification model. However, even though the problem can be narrowed down to a supervised regression problem by the given dataset, there remain a myriad of different approaches and algorithms. Moreover, since the dual analyte sensor is sensitive to two analytes simultaneously, the algorithm should reflect that and output both analyte information at the same time. Therefore, we decided to build a multi-layered ML model that first finds the pH that best fits a given spectral response of the dual analyte sensor and then iteratively finds a solution for dissolved O_2_. The reason for this order of the multi-layered model was that the dual analyte sensor appears to be more sensitive to changes in O_2_ concentration, and cross-interactions that occur, such as FRET or alike, impact the overall sensor response more than changes in pH (see Fig. 3). Furthermore, please note that we used the absolute fluorescence spectra instead of the ratiometric ones, as is usually the case in optical chemical imaging [12].

To now find the best ML regression algorithm, we have assessed different options and determined the performance of the overall model for the given data using different loss functions. For applied ML, the choice of the loss function can be very crucial and can lead to the favoring of different algorithms depending on where the focus lies for a given problem, i.e., whether, for example, accuracy is more relevant than sensitivity or selectivity of the sensor. One common way of describing the performance of a regression model is to determine its accuracy and dispersion in terms of mean absolute error (MAE) and root mean square error (RMSE), respectively [30]. However, while the performance measures of the dataset describe the overall performance of the algorithm for a given set, one cannot rule out that the data carry a hidden bias to which the algorithm mainly responds and trains. Thus, to prevent overfitting, the dataset is typically split into training and validation datasets. The former is used to train the model and describe the overall model performance, while the latter is used to comprehensively describe its performance on a dataset it has never seen before. Splitting the entire dataset is done using the standard split function of the Python package *lmfit*. This split function divides the dataset into random subsets according to a user-defined ratio, in our case, a ratio of 80:20. At first glance, this may seem counterintuitive compared to conventional validation tests where individual calibration points are removed for validation. However, the ML model is not based on one single deduced fit function and should thus be validated randomly over the entire calibration range. To find the optimal regression algorithm, all performance measures should be as low as possible. Tables 1 and 2 give an overview of the performance measures of the different ML algorithms and for each individual analyte.

**Table 1:** Performance of different ML regression algorithms assessed for training data as well as for validation data for the separate identification of pH

	Training – MAE	Training – RMSE	Validation – MAE	Validation – RMSE
Linear regression	0.395	0.533	0.462	0.639
Lasso regression	1.301	1.562	1.379	1.658
Ridge regression	0.395	0.533	0.462	0.639
Logistic regression	10.469	42.146	39.892	86.761
Random forest regression	0.104	0.171	0.285	0.465
Support-vector machine regression	0.230	0.437	0.596	0.884
***K***-nearest neighbors regression	0.986	1.274	1.461	1.854
Decision tree regression	0.000	0.000	0.283	0.640
Xgboost regression	0.025	0.034	0.311	0.444

**Table 2:** Performance of different ML regression algorithms assessed for training data as well as for validation data for the separate identification of dissolved O_2_

	Training – MAE	Training – RMSE	Validation – MAE	Validation – RMSE
Linear regression	18.106	23.444	18.507	24.092
Lasso regression	18.824	23.696	19.017	24.040
Ridge regression	18.111	23.444	18.511	24.091
Logistic regression	184.928	859.648	335.046	1244.190
Random forest Regression	1.491	3.379	3.680	7.839
Support-vector machine regression	27.695	39.443	30.823	43.895
***K***-nearest neighbors regression	14.885	23.666	23.162	35.234
Decision tree regression	0.000	0.000	2.556	10.865
Xgboost regression	0.642	0.998	4.474	7.886

As can be seen from Tables 1 and 2, there is not one regression algorithm that is best suited and provides optimal results for both analytes. However, the algorithms that perform best for both the training data and the validation data are the following regressors: decision tree (DT), random forest (RF), and XGBoost (XGB). Consequently, these three regressors were selected as potential candidates for the ML model, and their respective parameters were further optimized.

#### Model optimization

To fine-tune the algorithm and optimize its performance, there are several set screws that define the algorithm, control its learning process, and constrain the algorithm in minimizing a predefined loss function. These so-called hyperparameters can be optimized in a process called hyperparameter optimization (HPO). This has been done for all three potential candidates and each analyte. Traditional approaches to HPO are either a parameter sweep, in which parameters are optimized by comprehensively enumerating all combinations over a manually specified subset of the hyperparameter space or a random search, in which a subset of parameter combinations is randomly defined [38]. The supplemental information provides a detailed summary of the HPO process of all three algorithms and both analytes, while Table 3 summarizes the final performance of the optimized algorithms using the same performance measures (MAE and RMSE) as described previously. Please note that the attached Excel file contains a detailed summary of all HPO processes, intended as a guide for readers new to ML modeling to be able to replicate the steps for model optimization. The Word document provides a summary of the most important intermediate results for a quick overview. Note that the Excel file provides very detailed information on the HPO process.

**Table 3:** Performance of ML regression algorithms optimized in an HPO process. Performance is assessed for both the training data and for the validation data for the separate identification of pH and O_2_, respectively

		Training – MAE	Training – RMSE	Validation – MAE	Validation – RMSE
Random forest regression	pH	0.133	0.177	0.205	0.290
	O_2_	0.738	1.934	1.439	3.718
Decision tree regression	pH	0.201	0.356	0.251	0.448
	O_2_	0.536	2.681	0.997	4.943
Xgboost regression	pH	0.008	0.011	0.170	0.271
	O_2_	0.585	1.068	1.668	4.541

As shown in Table 3, the optimal regression algorithm for pH prediction is the XGBoost regression algorithm, a scalable decision tree-based ensemble ML algorithm that uses a gradient boosting framework and provides a parallel tree boosting [39]. This is not surprising, since for small- to medium-sized structured data, XGBoost like all decision tree-based algorithms is considered to be the best performing. While the performance measures for pH prediction are clearly in favor of the XGBoost regression algorithm, this is less clear for dissolved O_2_ prediction. We have therefore decided to use the XGBoost regression algorithm for the prediction of dissolved O_2_ as well. Table 4 summarizes the optimized hyperparameter for each analyte yielding the performance metrics described before (Table 3).

**Table 4:** Optimized hyperparameter for each XGBoost regressor

	pH prediction (1st layer)	O_2_ prediction (2nd and 3rd layer)
n_estimators	250	250
min_child_weight	3	5
max_depth	9	7
learning_rate	0.05	0.05

#### Final ML model and model validation

Upon performing several optimization and screening procedures, the final model for simultaneous detection of pH and dissolved O_2_ using optical chemical sensors now consists of a two-layer ML model based on XGBoost algorithms. First, the pH value is predicted and, subsequently the O_2_ concentration with conditional knowledge of the pH value. However, since the prediction of dissolved O_2_ appeared to be rather uncertain, with some outliers and larger uncertainties, an additional ML layer using an XGBoost regression algorithm was used to iteratively optimize the O_2_ prediction. Thus, the final ML model includes three XGBoost layers for pH and O_2_ prediction. The final algorithm can be downloaded from GitHub (github.com/silviaelisabeth/ML_for_pHandO2) and is freely available.

As mentioned several times in this publication, the validation of an ML model is crucial in the building process to ensure its accuracy and to prevent any bias in the dataset. Besides validation by one-time sub-sampling, as performed previously, another option is cross-validation [36]. Cross-validation is a resampling method that uses either individual samples or a larger subsample from the entire dataset to validate a model on different iterations. During the validation process, the subsamples are removed from the training dataset to avoid a prediction bias. Due to this, however, cross-validation requires more computational power than validation by one-time sub-sampling; but it also assesses the performance of the model much more accurately. An extremely accurate but, due to the large size of our dataset, computational intense cross-validation would be a leave-one-out cross-validation in which all possible combinations are trained and tested. Consequently, a good balance between these two approaches, validation by one-time sub-sampling or validation by leave-one-out cross-validation, would thus be *k*-fold cross-validation, a non-exhaustive cross-validation in which the dataset is randomly partitioned in *k* equally sized subsamples. While one of the *k* subsamples is used as validation data, the remaining subsamples are used as training data. This process is then repeated *k* times. In ML modeling, it is common practice to perform *10-*fold cross-validation [36]. Subsequent to cross-validation, MAE and RMSE can be determined as performance metrics as described before. A summary of the validation process for the ML model is listed in Table 5, while detailed information about the variance of predicted and target pH and dissolved O_2_, respectively, can be found in the supplemental information. In order to illustrate the benefits of iterative O_2_ prediction, we listed in Table 5 the performance metrics for the initial O_2_ prediction as well as the performance metrics after adding the additional layer for iterative O_2_ prediction. Please note that *10-*fold cross-validation indicates the minimum performance of a model, whereas the true performance of the model is better and achieves lower dispersions.

**Table 5:** Performance metrics of the multi-layered ML model for simultaneous prediction of pH and dissolved O_2_ concentration determined based on a *10-*fold cross-validation

*10-*fold cross-validation	Mean absolute error (MAE)	Root mean square error (RMSE)
pH prediction	1.96·10^−1^	4.42·10^−1^
O_2_ prediction, 1st layer	1.57	1.25
O_2_ prediction, 2nd layer	4.50·10^−2^	2.12·10^−1^

**Fig. 5:**
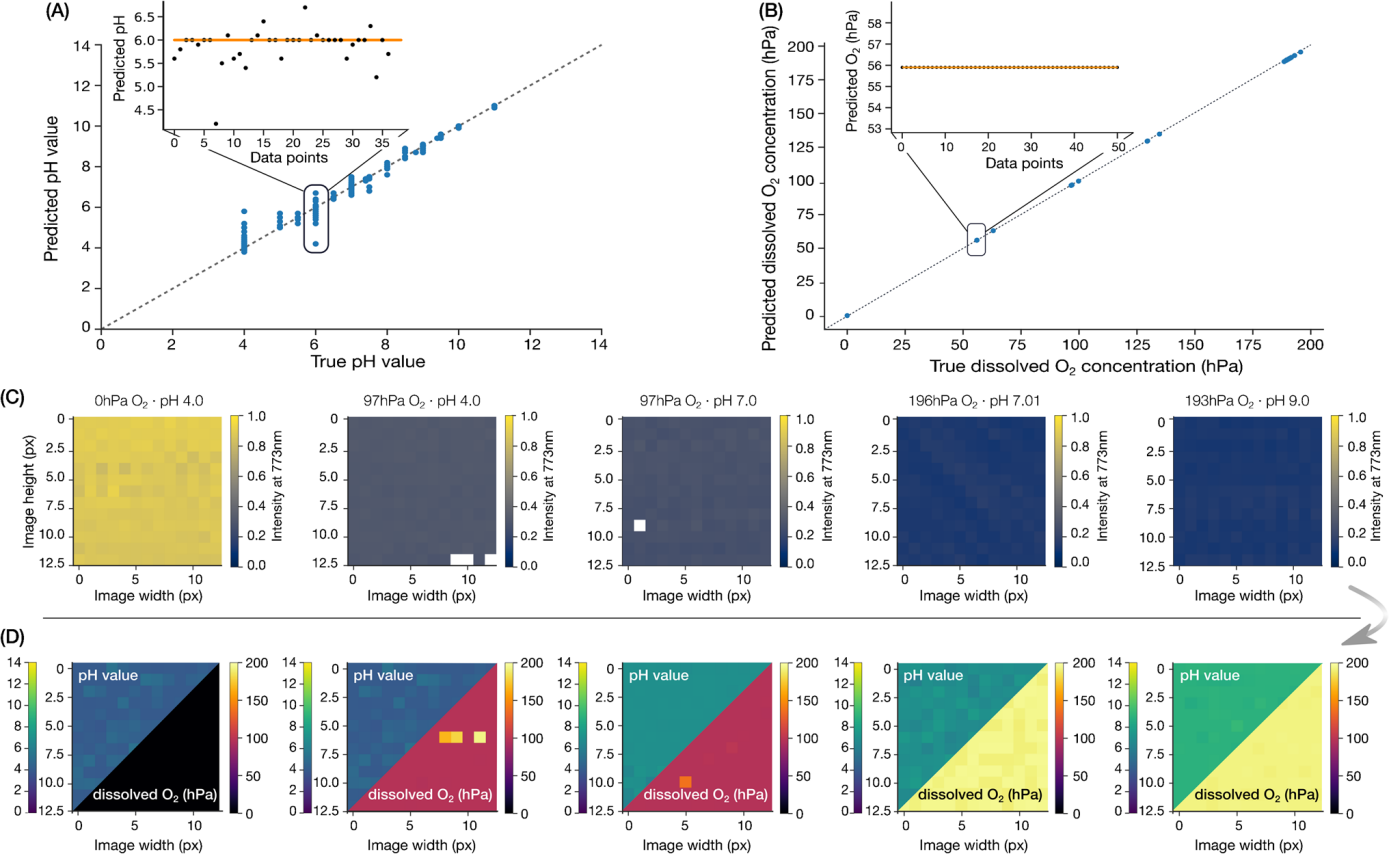
Overall model performance for predicting the pH (**A**) and dissolved O_2_ concentration (**B**), respectively, assessed against test data that the algorithm has never seen before. The main plot compares the predicted and the respective target values for the entire calibration range using the multi-layered ML model based on XGBoost. The insets of each panel display the dispersion around the target value as black dotted markers. The target value is indicated as an orange solid line. **C** and **D** display examples of optode images before and after data analysis. **C** shows the absolute fluorescence intensity of the dual analyte optode is visualized at 773 nm, whereas **D** shows the chemical images in which the absolute fluorescence intensity has been translated into the corresponding pH and O_2_ concentration (in hPa) to represent them in each pixel

As can be seen from Table 5, the iterative process of O_2_ prediction leads to particularly small performance measures with a mean absolute error of < 4.50·10^−2^ (compared to < 1.57 for non-iterative O_2_ prediction) and a root mean squared error of < 2.12·10^−1^ (< 1.25 previously). This reduces the uncertainty of the O_2_ prediction on average to the third decimal. As described in the supplemental information (cf. Section 4), the iterative approach for prediction of dissolved O_2_ helps circumvent potential reabsorption and interference artifacts. In contrast, the performance measures for pH prediction are significantly worse, with mean absolute error and root mean square error of < 1.96·10^−1^ and < 4.42·10^−1^, respectively. Thus, a prediction of pH is on average less accurate with this model and varies in the first decimal. A detailed discussion of the deviation of the predicted pH values compared to the target pH values can be found in the supplemental information (see Section 3). As shown in Fig. 5, as well as in Figure·S2 and Figure·S3, the deviations occur mainly at lower pH values when reabsorption effects and indicator interactions are more dominant. Furthermore, it should be noted that although the calibration is performed over the entire pH range, the dynamic range of the pH sensing layer is, however, limited to a range of ± 2 pH units around the pK_a_ value, i.e., a range between 5 and 9. pH values outside this range are not considered physically reasonable but invalid, even if it would be possible for the ML algorithm to find a pattern. However, so far, we have not performed any additional experiments to investigate the limitations of the ML model in this regard and therefore recommend using the pH sensing layer only in the known dynamic range. Moreover, where necessary, the performance of the pH prediction could be optimized with an additional model layer, as has been done for the prediction of dissolved O_2_. However, it should be emphasized again that these values are minimum values; the actual performance of the model is better.

## Conclusion

We have developed a novel approach to multi-analyte optical chemical imaging in 2D using a machine learning model and outlined the building process of the multi-layered ML model for complex and coupled data. While the dual analyte optode calibration data for simultaneous imaging of pH and dissolved O_2_ cannot be explained by conventional multivariate analysis methods, machine learning algorithms have proven useful. Consequently, we were able to build a three-layered model with individual pH and iterative O_2_ prediction based on a decision tree-based algorithm (the so-called XGBoost). Figure 5C–D illustrates the conversion of the absolute fluorescence intensity emitted by the optode to the corresponding pH and concentration of dissolved O_2_ in each pixel. While the dissolved O_2_ can thus be predicted with an average error of < 0.045 (MAE) and < 0.212 (RMSE), pH is predicted with an average error of < 0.196 (MAE) or < 0.442 (RMSE), respectively. In other words, the iterative prediction of dissolved O_2_ works excellently, while the pH prediction can be improved, if necessary, as shown in the discussion.

While our contribution demonstrates the advantages of ML models for nested and intercoupled datasets that cannot be solved with conventional statistical models, we also highlighted the risks during the ML model process. For each process step during ML model development, we highlighted different risks that researchers should consider when building their own ML model, including data preparation (outlier test vs. dimension reduction), establishing a balanced training dataset and identifying and validating an appropriate ML model for the question at hand. Researchers need to be aware that while it is inherent to ML models to find patterns, it is our responsibility to not indulge into p-hacking or data dredging and follow patterns that in reality do not exist.

Despite the risks that come with ML modeling, we should dare and bring data analysis out of its shadowy existence. We should give it due attention if we want to advance multi-analyte imaging. In particular from a practical aspect, this approach appears very appealing. ML can help construct multi-analyte sensors using already existing indicators and circumvents the need to find indicators that have limited spectral overlap or other types of interactions. Converting acquired images into quantitative data is often cumbersome and not simple, especially when additional signal deconvolution is required. ML algorithms clearly show advantages in deciphering intercoupled datasets with high dimensionality and complexity, where human imagination and conventional methods fail in finding the underlying correlations. However, we must not blindly use any ML algorithm but also be aware of the possible biases and risks when setting up training and validation data.

## Supplementary Information

Below is the link to the electronic supplementary material.

Supplementary file1 (DOCX 897 KB)

Supplementary file2 (PDF 2463 KB)

## Abbreviations

ML: Machine learning
RoI: Region of interest
MAE: Mean absolute error
RMSE: Root mean square error
XGB: XGBoost regression algorithm
DT: Decision tree regression algorithm
RF: Random forest regression algorithm
HPO: Hyperparameter optimization
