# Supplementary material for: Machine learning for optical chemical multi-analyte imaging: Why we should dare and why it’s not without risks
Source: Anal Bioanal Chem. 2023 Apr 18;415(14):2749–61. doi: 10.1007/s00216-023-04678-8 (PMC10185573; doi:10.1007/s00216-023-04678-8)
Supplement: Supplementary file 1 — Supplementary file1 (DOCX 897 KB) [file 216_2023_4678_MOESM1_ESM.docx]

Machine Learning for Optical Chemical Multi-Analyte Imaging | Why we should dare and why it’s not without risks

Silvia E. Zieger and Klaus Koren*

Aarhus University Centre for Water Technology (WATEC), Department of Biology, Section for Microbiology, Aarhus University, 8000 Aarhus C, Denmark
* Corresponding author: [klaus.koren@bio.au.dk](mailto:klaus.koren@bio.au.dk)

Table of Contents

[1. Hyperparameter Optimization (HPO) of Potential Regression Algorithms for pH and dissolved O_2_ 2](#_Toc130200438)

[1.1. HPO of Potential Regression Algorithms for pH 2](#_Toc130200439)

[1.2. HPO of Potential Regression Algorithms for dissolved O_2_ 4](#_Toc130200440)

[2. Visualization of the individual model layers 6](#_Toc130200441)

[3. Cross-check of target and predicted pH value for the ML model 7](#_Toc130200442)

[4. Cross-check of target and predicted O_2_ concentration value for the ML model 8](#_Toc130200443)

## 1. Hyperparameter Optimization (HPO) of Potential Regression Algorithms for pH and dissolved O_2_

Hyperparameters are critical tuning parameters that control the learning process of machine learning models. To optimize these parameters, a combination of a naïve approach and random grid search is commonly used. The naïve approach provides an initial understanding of how a specific parameter affects the overall performance, while grid search fine-tunes the hyperparameters. However, the exact values obtained through this process are not always reproducible as they depend on the subset of training and validation data selected. Nevertheless, the direction of hyperparameter optimization is reproducible. To determine the model's true performance, the determination of performance should be repeated several times and an average taken. It's essential to analyze each hyperparameter individually while keeping other parameters at their default values.

An Excel file with a detailed summary of all processes performed for hyperparameter optimization (HPO) is included as an additional supporting information document. The file contains individual sheets for each HPO process for pH or dissolved O_2_ with the three selected regression algorithms (Random Forest, Decision Tree, and XGBoost). Each sheet includes plots that display the algorithm’s overall performance for different hyperparameter values, with the optimal parameter defined by the first "kink" in the optimization curve. While the Excel file provides a detailed summary, we also provide a brief summary of the HPO outcomes here.

### 1.1. HPO of Potential Regression Algorithms for pH

While the naïve approach to HPO is a manual approach by trying out different values for a certain hyperparameter. This provides the user with an initial idea of the impact of a specific parameter on the overall (benchmark) performance and the general tendency. The grid search has been used subsequently to fine-tune the hyperparameters.

#### 1.1.1. HPO of Random Forest

Naïve approach

The naïve approach to hyperparameter optimization is a parameter sweep, i.e., an exhaustive search of a manually specified subset of possible values of the hyperparameter. For each possible value of the hyperparameters, the performance parameters (MAE and RMSE) are determined. To decide which value yields sufficient performance, the threshold is set where the performance curve has a sharp bend. The data as well as the respective figures are summarized in the supplemental excel sheet *RandomForest_pH.*

The hyperparameters, optimized in this naïve approach, result in an overall model performance of the random forest regressor of < 0.28 (MAE) and < 0.38 (RMSE) for the training data and < 0.32 (MAE) and < 0.43 (RMSE) for the validation data, respectively.

RandomGridSearch

The results from the naïve approach were used in a random grid search function in which a certain search space for the individual hyperparameters is defined as a search grid. The performance is assessed in terms of MAE and RMSE as it was done before. The search function returns the best parameter combination resulting in the best model performance. In the case of pH, the random forest regressor worked best with the following hyperparameters:

- n_estimators 100
- min_weight_fraction_leaf 0
- min_samples_split 3
- min_samples_leaf 4
- max_leaf_nodes 90
- max_features 10
- max_depth 30
- bootstrap FALSE

These hyperparameters result in an overall model performance of the random forest regressor of < 0.133 (MAE) and < 0.177 (RMSE) for the training data and < 0.205 (MAE) and < 0.290 (RMSE) for the validation data, respectively.

#### 1.1.2. HPO of Decision Tree

Naïve approach

Same as it was done for the random forest regressor, the hyperparameters were optimized for the pH data set using a decision tree regression approach. The individual optimization is summarized in the supplemental excel sheet *DecisionTree_pH*. The naïve results in hyperparameters that allow an overall model performance of the decision tree regressor of < 0.53 (MAE) and < 0.74 (RMSE) for the training data and < 0.57 (MAE) and < 0.81 (RMSE) for the validation data, respectively.

RandomGridSearch

The results from the naïve approach were used in a random grid search function and in the case of the pH prediction, the decision tree regressor worked best with the following hyperparameters:

- splitter best
- min_weight_fraction_leaf 0
- min_samples_leaf 2
- max_leaf_nodes 70
- max_features None
- max_depth 6

The optimized hyperparameters result then in an overall model performance of the decision tree regressor of < 0.201 (MAE) and < 0.356 (RMSE) for the training data and < 0.251 (MAE) and < 0.448 (RMSE) for the validation data, respectively. As can be seen, the decision tree performs better than the random forest regressor on both the training and validation data.

#### 1.1.3. HPO of XGBoost

Naïve approach

The hyperparameters were optimized for the pH data set using a scalable decision tree regression approach called XGBoost. The individual optimization is summarized in the supplemental excel sheet *XGBoost_pH*. With the naïve approach, hyperparameters have been found that result in an overall model performance of the XGBoost regressor of < 0.52 (MAE) and < 0.62 (RMSE) for the training data and < 0.56 (MAE) and < 0.67 (RMSE) for the validation data, respectively.

RandomGridSearch

Again, results from the naïve approach were used in a random grid search function, and in the case of the pH prediction, the XGBoost regressor worked best with the following hyperparameters:

- n_estimator 250
- min_child_weight 3
- max_depth 9
- learning_rate 0.05

Thus, the hyperparameters result in an overall model performance of the XGBoost regressor of < 0.008 (MAE) and < 0.011 (RMSE) for the training data and < 0.170 (MAE) and < 0.271 (RMSE) for the validation data, respectively. Consequently, when comparing the performance parameters of the different approaches, the XGBoost regressor works best for the pH prediction and is therefore used for the ultimate ML model.

### 1.2. HPO of Potential Regression Algorithms for dissolved O_2_

While the naïve approach to HPO is a manual approach by trying out different values for a certain hyperparameter. This provides the user with an initial idea of the impact of a specific parameter on the overall (benchmark) performance and the general tendency. The grid search has been used subsequently to fine-tune the hyperparameters. It should be noted, however, that the exact values are not reproducible as they depend on the selected training and test data; however, the direction of the specific hyperparameter optimization is reproducible. In addition, the determination of performance must be repeated several times to determine the average (true) performance of the model. Each hyperparameter has been analyzed individually while other hyperparameters have been set to their default values.

#### 1.2.1. HPO of Random Forest

Naïve approach

The naïve approach to hyperparameter optimization is a parameter sweep, i.e., an exhaustive search of a manually specified subset of possible values of the hyperparameter. For each possible value of the hyperparameters, the performance parameters (MAE and RMSE) are determined. To decide which value yields sufficient performance, the threshold is set where the performance curve has a sharp bend. The data as well as the respective figures are summarized in the supplemental excel sheet *RandomForest_O2.*

The hyperparameters, optimized in the naïve approach, result in an overall model performance of the random forest regressor of < 3.12 (MAE) and < 5.49 (RMSE) for the training data and < 2.89 (MAE) and < 5.39 (RMSE) for the validation data, respectively.

RandomGridSearch

The results from the naïve approach were subsequently used in a random grid search function in which a certain search space for the individual hyperparameters is defined as a search grid. The performance is assessed in terms of MAE and RMSE as it was done before. The search function returns the best parameter combination resulting in the best model performance. In the case of dissolved oxygen, the random forest regressor worked best with the following hyperparameters:

- n_estimators 100
- min_weight_fraction_leaf 0
- min_samples_split 3
- min_samples_leaf 4
- max_leaf_nodes 90
- max_features 10
- max_depth 0
- bootstrap FALSE

These hyperparameters result in an overall model performance of the random forest regressor of < 0.738 (MAE) and < 1.934 (RMSE) for the training data and < 1.439 (MAE) and < 3.718 (RMSE) for the validation data, respectively.

#### 1.2.2. HPO of Decision Tree

Naïve approach

The naïve approach of the hyperparameter optimization for dissolved oxygen is summarized in the supplemental excel sheet *DecisionTree_O2*. As benchmark performance, the naïve resulted in hyperparameters that allow an overall model performance of < 4.84 (MAE) and < 10.28 (RMSE) for the training data and < 4.95 (MAE) and < 10.47 (RMSE) for the validation data, respectively.

RandomGridSearch

The results from the naïve approach were used in a random grid search function and in the case of the dissolved O_2_ prediction, the decision tree regressor worked best with the following hyperparameters:

- splitter best
- min_weight_fraction_leaf 0
- min_samples_leaf 3
- max_leaf_nodes 100
- max_features 7
- max_depth 10

The optimized hyperparameters result then in an overall model performance of the decision tree regressor of < 0.536 (MAE) and < 2.681 (RMSE) for the training data and < 0.997 (MAE) and < 4.943 (RMSE) for the validation data, respectively. As can be seen, the random forest performs better than the decision tree regressor in almost all performance criteria for both the training and validation data. Only the MAE for test data was better when using the decision tree regressor.

#### 1.2.3. HPO of XGBoost

Naïve approach

The hyperparameters were optimized for the dissolved oxygen data set using a scalable decision tree regression approach called XGBoost. The individual optimization is summarized in the supplemental excel sheet *XGBoost_O2*. With the naïve approach, hyperparameters have been found that result in an overall model performance of the XGBoost regressor of < 5.78 (MAE) and < 6.50 (RMSE) for the training data and < 6.08 (MAE) and < 7.76 (RMSE) for the validation data, respectively.

RandomGridSearch

Again, results from the naïve approach were used in a random grid search function, and in the case of the dissolved O_2_ prediction, the XGBoost regressor worked best with the following hyperparameters:

- n_estimator 250
- min_child_weight 5
- max_depth 7
- learning_rate 0.05

Thus, the hyperparameters result in an overall model performance of the XGBoost regressor of < 0.585 (MAE) and < 1.068 (RMSE) for the training data and < 1.668 (MAE) and < 4.541 (RMSE) for the validation data, respectively. Consequently, when comparing the performance parameters of the different approaches, the XGBoost regressor works best for the dissolved O_2_ prediction on the training data. On the test data, it seems that the random forest regressor might perform better. However, in order to optimize the overall model performance, we decided on the XGBoost and added an additional (iterative) layer for O_2_ prediction.

## 2. Visualization of the individual model layers

For interpretability of the individual model layers, we display here the first decision tree of the trained XGBoost models. Please note, that XGBoost consists of an ensemble of various trees. Hence, a full visualization of all trees is not possible. However, in order to provide an idea of which features of the data set are more relevant, we display the first tree model. In the model, each node resembles the feature the algorithm identified as most relevant for the decision as well as its value. The features are labeled with indices in ascending order. At the end of each branch, the leaf node is displayed.


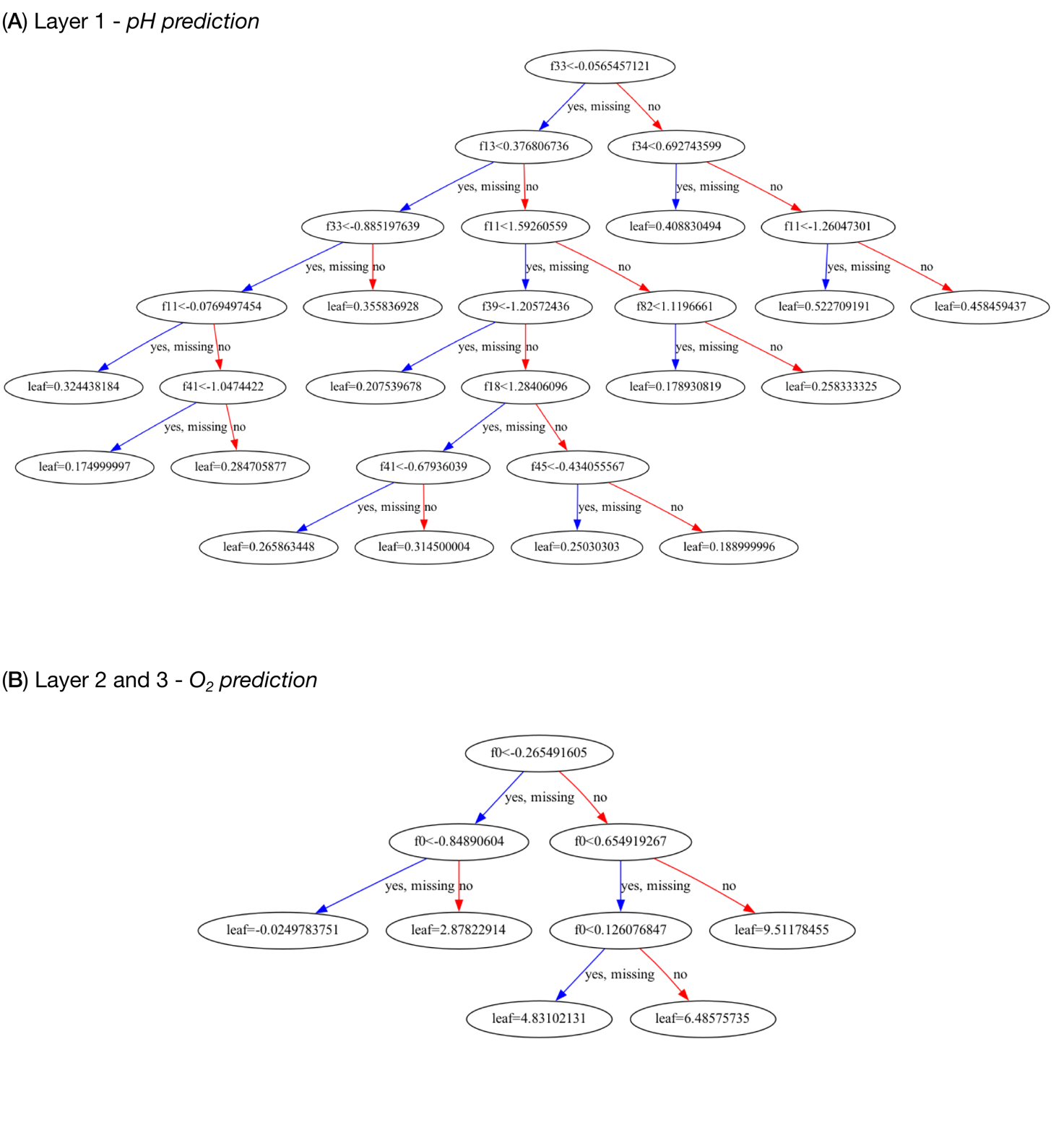


Figure·S 1 Visualization of the first decision tree of each model layer for pH prediction (A) and dissolved O_2_ prediction (B) using individually trained XGBoost models. In the tree model, each node resembles the feature the algorithm identified as most relevant for the decision as well as its value. The features are labeled with indices in ascending order. At the end of each branch, the leaf node is displayed.

## 3. Cross-check of target and predicted pH value for the ML model

Figure·S 2 Comparison of predicted and target pH value upon application of the multi-layered ML model based on XGBoost. While the main plot displays all samples over the entire calibration range, the inset displays the dispersion, around the target value for calibration point pH 6 in black dotted markers. The target pH is shown as a solid orange line. Panel (A) displays the training data, while panel (B) displays the validation data.

As can be seen from Figure·S 2, the dispersion of the predicted pH values around the target pH values is generally small for the training data, with a few exceptions. As expected, the dispersion of the predicted pH values around the target pH values is larger for the test data that the ML model has not seen before. In particular, at lower pH values, the dispersion is larger. This could be explained by the larger reabsorption effects between the two sensing layers as described previously. It should be noted, however, that although calibration is performed over the entire pH range, the dynamic range of the pH sensing layer is still limited to a range of ± 2 pH units around the pKa value, i.e., to a range between 5-9. pH values outside this range are not valid and are not considered physically reasonable.

## 4. Cross-check of target and predicted O_2_ concentration value for the ML model

Figure·S 3 Comparison of predicted and target O_2_ concentration upon application of the multi-layered ML model based on XGBoost. While the main plot in panels (A) and (B) displays all data samples over the entire calibration range, the insets display the dispersion, around the target value for calibration point 6% O_2_ as black dotted markers. The target O_2_ concentration is shown as a solid orange line. Panel (A) displays the training data and panel (B) displays the validation data.

As can be seen from Figure·S 3, the dispersion of the predicted concentration of dissolved O_2_ around the target O_2_ values for the training data is generally low, which is also resembled in the very low performance parameters MAE and RMSE (see main section of the publication). Due to the iterative approach of O_2_ prediction, the test data also display a very low deviation of the predicted values from the target values. In this case, the iterative approach apparently helps to circumvent possible reabsorption and interfering artifacts.
