# Supplementary material for: Machine learning for optical chemical multi-analyte imaging: Why we should dare and why it’s not without risks
Source: Anal Bioanal Chem. 2023 Apr 18;415(14):2749–61. doi: 10.1007/s00216-023-04678-8 (PMC10185573; doi:10.1007/s00216-023-04678-8)
Supplement: Supplementary file 2 — Supplementary file2 (PDF 2463 KB) [file 216_2023_4678_MOESM2_ESM.pdf]

Hyperparameter optimization for the pH data set using a Random Forest Regressor

(A) Naive approach

n_estimators	Training data set		Test data set	
	MAE	RMSE	MAE	RMSE
1	0.208	0.313	0.25	0.413
10	0.171	0.229	0.232	0.337
20	0.162	0.217	0.219	0.312
50	0.156	0.21	0.206	0.288
75	0.155	0.206	0.207	0.286
100	0.157	0.209	0.209	0.289
125	0.156	0.207	0.205	0.284
150	0.158	0.209	0.208	0.287
175	0.155	0.206	0.204	0.284
200	0.154	0.205	0.204	0.283

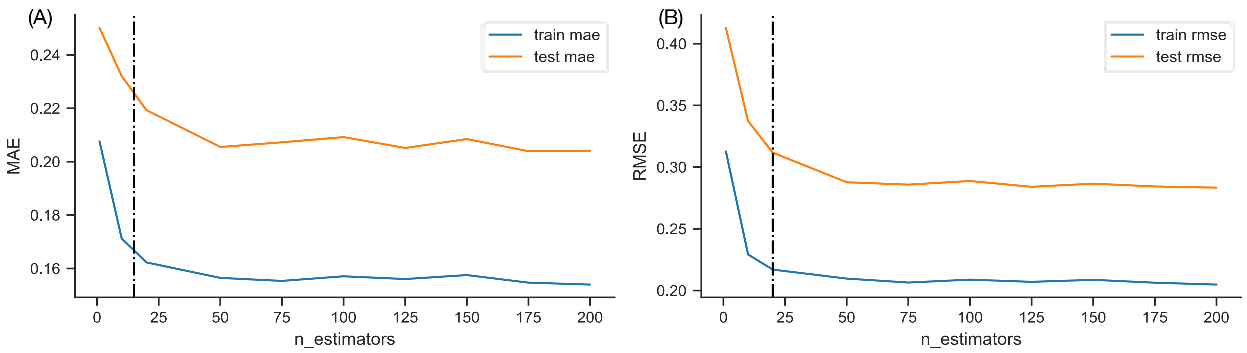

min_weight_fraction_leaf	Training data set		Test data set	
	MAE	RMSE	MAE	RMSE
0	0.166	0.223	0.231	0.324
0.1	0.448	0.636	0.504	0.739
0.2	0.564	0.775	0.628	0.893
0.3	0.85	1.052	0.889	1.145
0.4	0.831	1.097	0.901	1.193
0.5	0.89	1.119	0.905	1.177

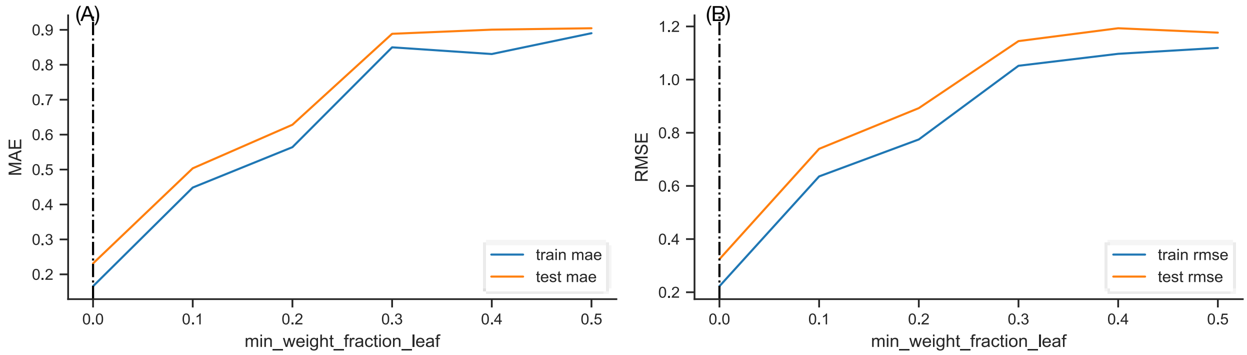

min_samples_split	Training data set		Test data set	
	MAE	RMSE	MAE	RMSE
0.1	0.327	0.477	0.389	0.574
0.25	0.442	0.628	0.519	0.744
0.5	0.672	0.871	0.695	0.925

0.75	0.747	0.987	0.801	1.078
1	0.738	0.981	0.781	1.064
2	0.16	0.219	0.217	0.302
3	0.16	0.215	0.215	0.303
4	0.162	0.22	0.215	0.305
5	0.169	0.225	0.228	0.321
7	0.164	0.223	0.214	0.3
10	0.162	0.223	0.215	0.319

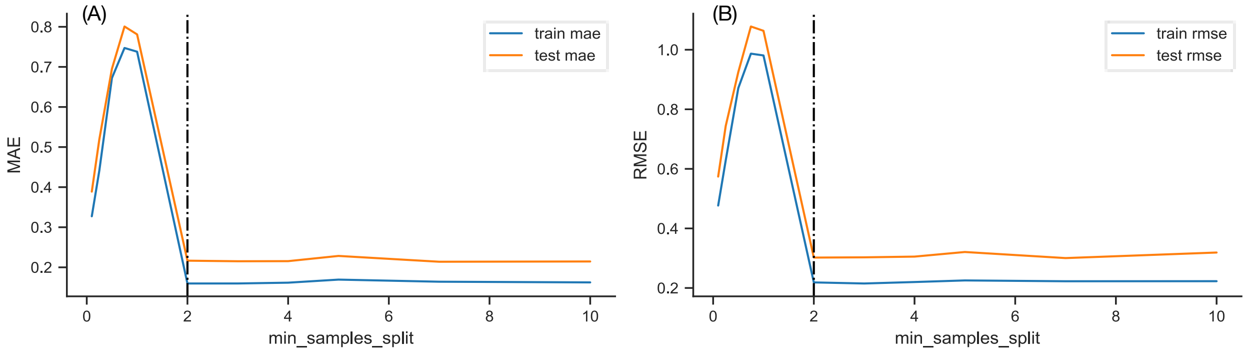

min_samples_leaf	Training data set		Test data set	
	MAE	RMSE	MAE	RMSE
1	0.163	0.218	0.219	0.306
3	0.169	0.228	0.229	0.313
4	0.165	0.221	0.221	0.306
5	0.164	0.22	0.208	0.29
6	0.16	0.22	0.2	0.289
7	0.162	0.224	0.221	0.319
8	0.164	0.226	0.22	0.321
9	0.173	0.234	0.216	0.3
10	0.167	0.23	0.222	0.323

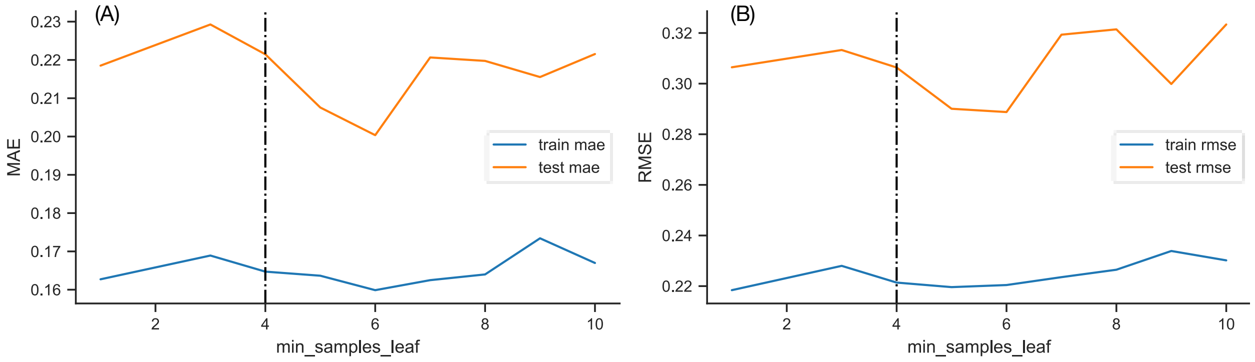

max_leaf_nodes	Training data set MAE	RMSE	Test data set MAE	RMSE
2	0.899	1.101	0.942	1.191
5	0.445	0.647	0.509	0.753
10	0.357	0.487	0.408	0.573
20	0.268	0.358	0.316	0.421
30	0.221	0.297	0.242	0.327
40	0.212	0.281	0.263	0.361
50	0.193	0.259	0.235	0.331
60	0.172	0.232	0.215	0.303
70	0.166	0.224	0.223	0.304
80	0.15	0.206	0.207	0.303
90	0.144	0.198	0.19	0.277
100	0.131	0.184	0.183	0.269
150	0.109	0.152	0.201	0.303
200	0.089	0.13	0.183	0.283

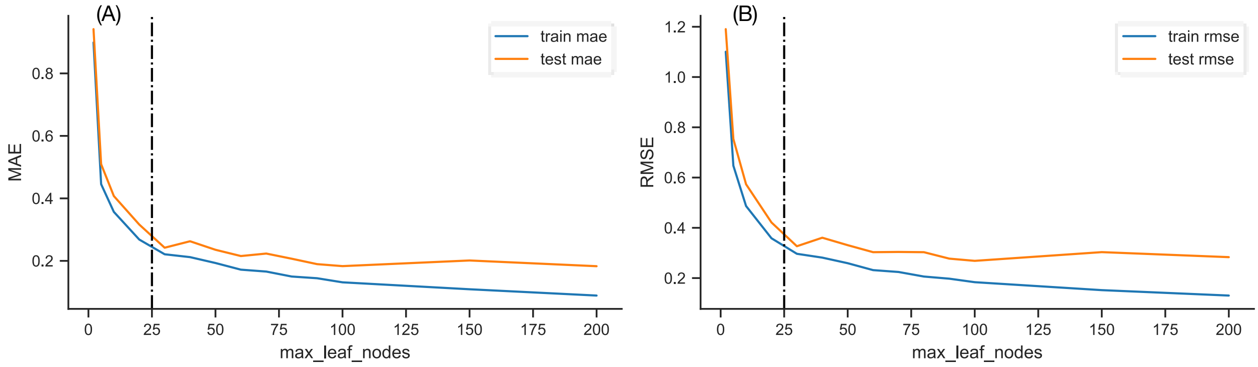

max_features	Training data set MAE	RMSE	Test data set MAE	RMSE
0.5	0.48	0.703	0.562	0.811
1	0.518	0.741	0.601	0.846
2	0.637	0.883	0.686	0.974
3	0.624	0.858	0.718	0.986
4	0.599	0.797	0.619	0.868
5	0.603	0.812	0.649	0.886
6	0.509	0.695	0.558	0.784
7	0.501	0.687	0.528	0.767
8	0.499	0.693	0.554	0.781
9	0.526	0.719	0.59	0.829
10	0.473	0.689	0.539	0.801
15	0.463	0.686	0.528	0.786
20	0.48	0.684	0.548	0.791
30	0.46	0.675	0.531	0.785

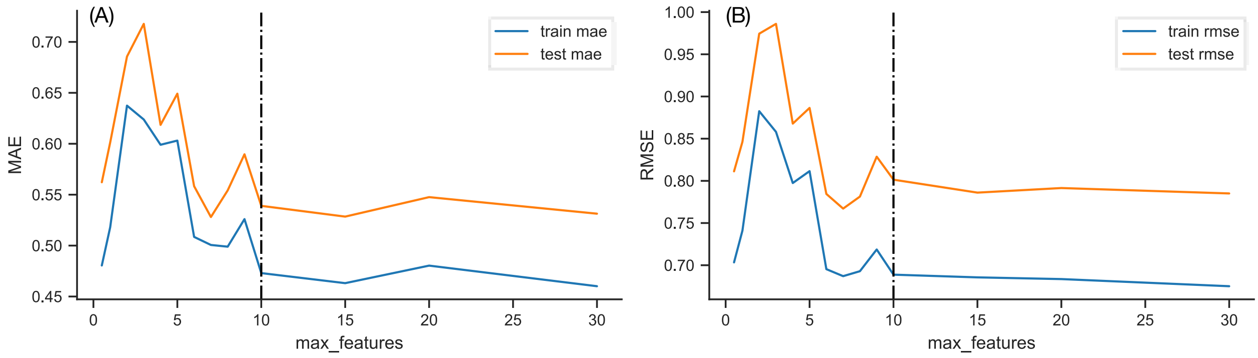

max_depth	Training data set MAE	Training data set RMSE	Test data set MAE	Test data set RMSE
1	0.78	1.013	0.822	1.111
2	0.549	0.759	0.598	0.853
3	0.498	0.7	0.571	0.832
4	0.48	0.682	0.549	0.793
5	0.483	0.678	0.54	0.77
6	0.484	0.686	0.55	0.793
7	0.489	0.687	0.541	0.789
8	0.494	0.695	0.537	0.777
9	0.511	0.702	0.561	0.792
10	0.465	0.669	0.528	0.775
15	0.495	0.684	0.555	0.78
20	0.505	0.71	0.566	0.811
30	0.493	0.698	0.556	0.818
40	0.505	0.691	0.559	0.795
50	0.5	0.698	0.559	0.813
60	0.484	0.694	0.539	0.789
70	0.473	0.669	0.525	0.764
80	0.492	0.695	0.544	0.795
90	0.484	0.672	0.544	0.767
100	0.493	0.664	0.543	0.764

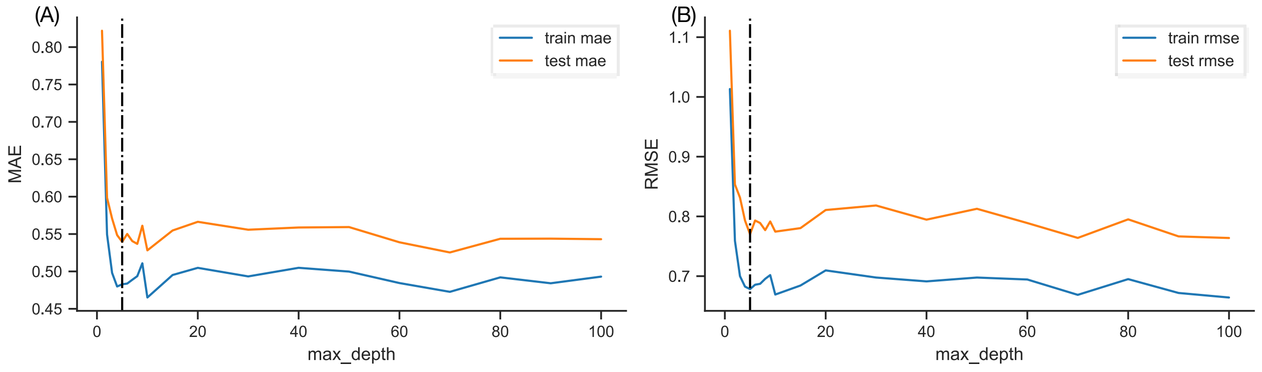

Bootstrap	Training data set MAE	Training data set RMSE	Test data set MAE	Test data set RMSE
TRUE	0.482	0.705	0.556	0.829
FALSE	0.516	0.714	0.591	0.828

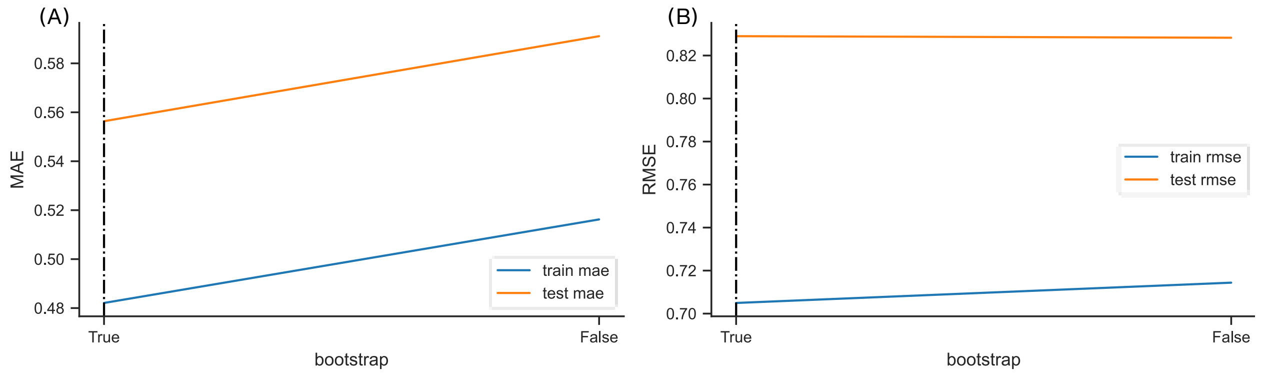

Hyperparameter optimization for the pH data set using a Decision Tree Regressor

(A) Naive approach

splitter	Training data set MAE	Training data set RMSE	Test data set MAE	Test data set RMSE
best	2.84E-16	1.00E-15	0.119	0.336
random	1.38E-16	6.14E-16	0.230	0.562

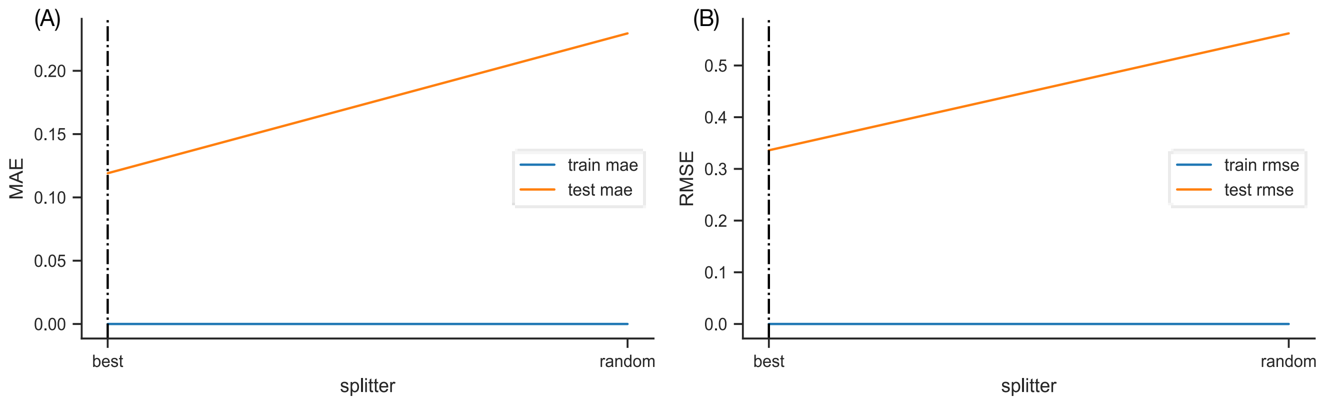

max_depth	Training data set mae	Training data set rmse	Test data set mae	Test data set rmse
1	0.969	1.162	1.012	1.242
3	0.480	0.663	0.537	0.744
5	0.258	0.424	0.269	0.431
7	0.135	0.290	0.161	0.343
9	0.074	0.205	0.136	0.328
11	0.035	0.138	0.129	0.343
15	0.003	0.029	0.127	0.346
20	5.07E-16	1.87E-15	0.138	0.370
25	4.37E-16	1.57E-15	0.126	0.339
30	2.83E-16	1.00E-15	0.120	0.339
50	2.84E-16	1.00E-15	0.122	0.348

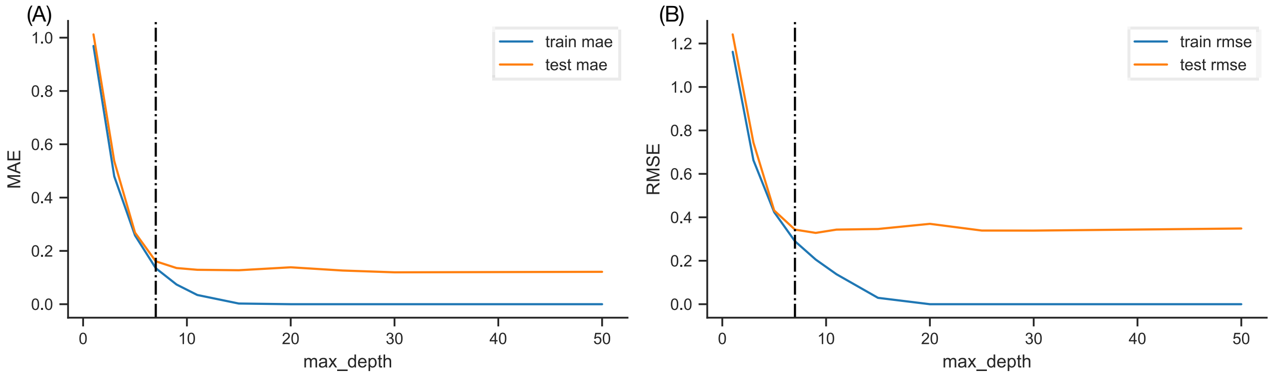

min_samples_leaf	Training data set mae	Training data set rmse	Test data set mae	Test data set rmse
0.5	0.980	1.184	1.013	1.250
0.75	1.667	2.004	1.734	2.083
1	0.135	0.290	0.157	0.340
1.5	1.667	2.004	1.734	2.083
2	0.137	0.291	0.158	0.341
3	0.139	0.294	0.162	0.341
4	0.140	0.295	0.158	0.336
5	0.142	0.297	0.165	0.343
6	0.145	0.300	0.164	0.346
7	0.147	0.302	0.174	0.357
8	0.148	0.303	0.175	0.358
9	0.150	0.305	0.179	0.357
10	0.153	0.307	0.176	0.352

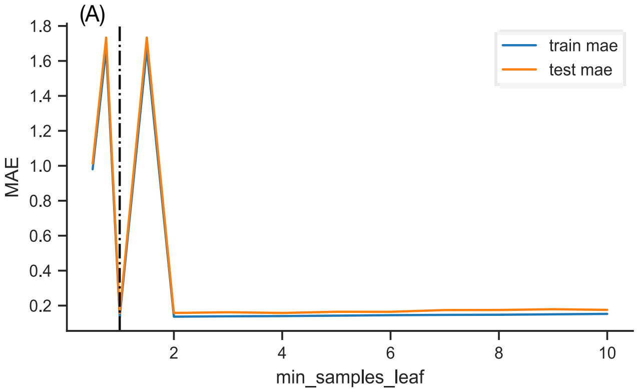

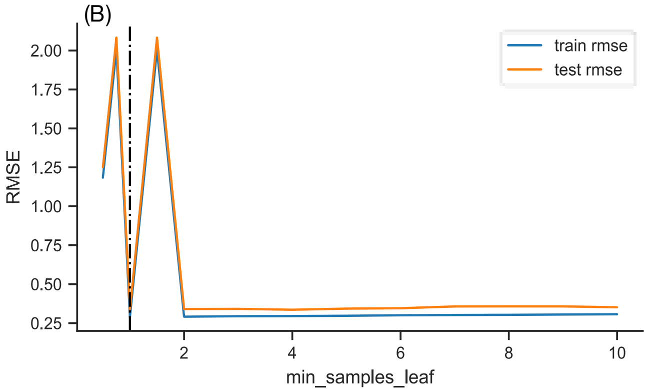

min_weight_fraction_leaf	Training data set mae	Training data set rmse	Test data set mae	Test data set rmse
0.0	0.135	0.290	0.152	0.330
0.1	0.491	0.668	0.561	0.776
0.2	0.614	0.805	0.685	0.894
0.3	0.969	1.162	1.012	1.242
0.4	0.969	1.162	1.012	1.242
0.5	0.980	1.184	1.013	1.250

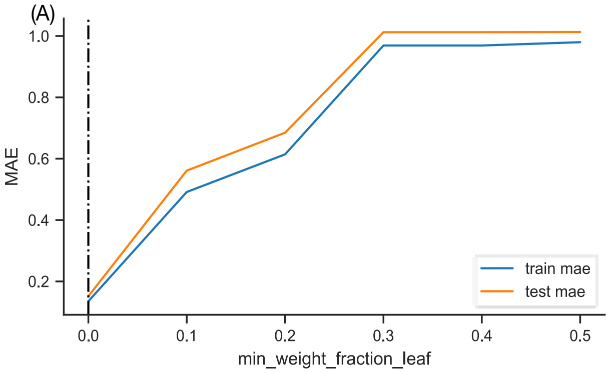

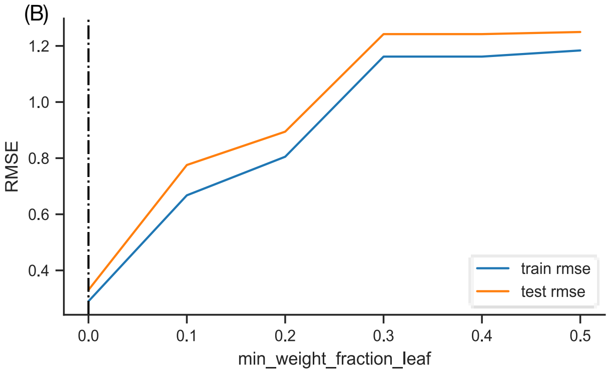

max_features	Training data set mae	rmse	Test data set mae	rmse
0.5	0.173	0.313	0.244	0.467
1	0.754	1.106	0.863	1.229
2	0.423	0.665	0.496	0.817
3	0.383	0.598	0.466	0.799
4	0.292	0.476	0.378	0.651
5	0.264	0.399	0.329	0.522
6	0.261	0.428	0.321	0.526
7	0.206	0.318	0.298	0.585
8	0.251	0.418	0.310	0.517
9	0.315	0.494	0.362	0.590
10	0.222	0.357	0.306	0.552
15	0.203	0.341	0.276	0.524
20	0.186	0.311	0.294	0.576
50	0.185	0.312	0.249	0.450
100	0.146	0.298	0.183	0.378
150	0.135	0.290	0.161	0.343

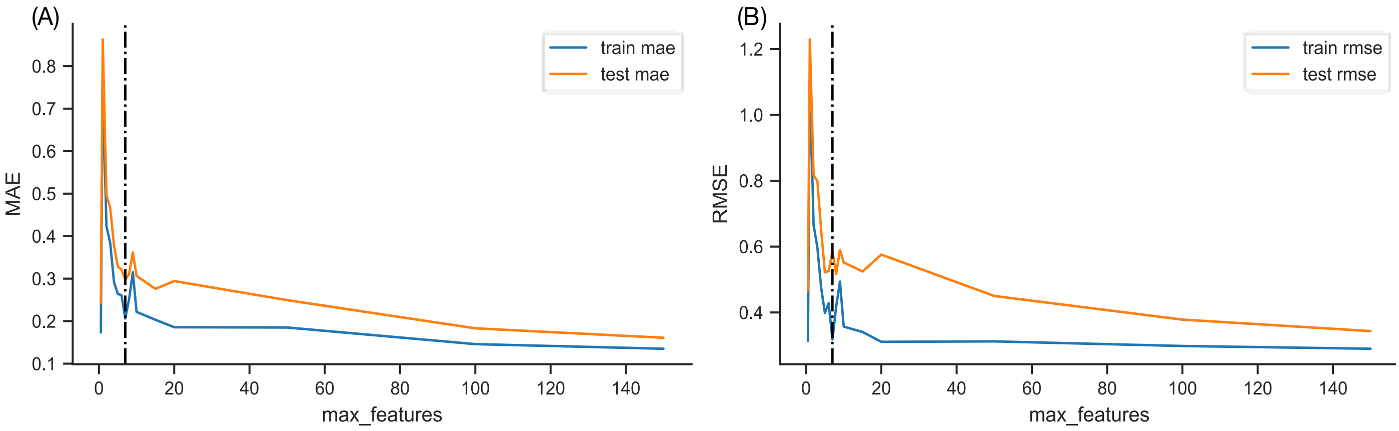

max_leaf_nodes	Training data set mae	rmse	Test data set mae	rmse
2	0.969	1.162	1.012	1.242
3	0.743	0.955	0.752	0.961
4	0.809	1.051	0.896	1.195
5	0.769	1.026	0.902	1.211
6	0.699	0.964	0.774	1.078
7	0.484	0.698	0.490	0.717
8	0.597	0.815	0.646	0.856
9	0.493	0.669	0.491	0.681
10	0.486	0.662	0.526	0.774
15	0.380	0.540	0.374	0.599
20	0.379	0.532	0.439	0.674
50	0.284	0.460	0.325	0.564
100	0.254	0.400	0.288	0.465
150	0.244	0.380	0.329	0.637

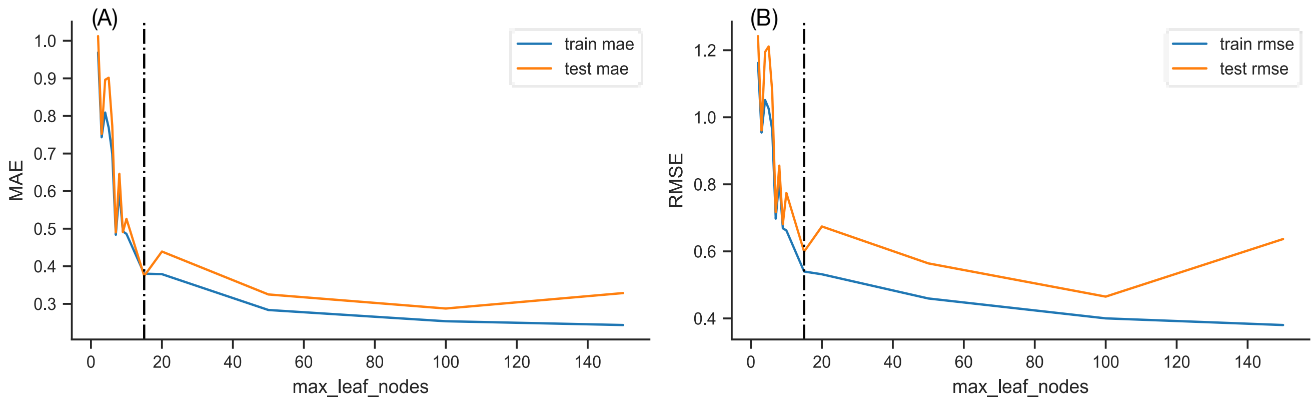

(B) Comparison naive approach vs RandomGridSearch

	splitter	max_features	max_depth	min_weight_	min_samp	max_leaf_nodes
**naive approach**	best	7	7	0	1	15
**(Random)GirdSearch**	best	None	6	0	2	70

performance of Decision Tree Regressor for pH data	Training data set		Test data set	
	MAE	RMSE	MAE	RMSE
**naive approach**	0.534	0.736	0.574	0.814
**(Random)GirdSearch**	0.201	0.356	0.251	0.448

Hyperparameter optimization for the pH data set using a XGBoost Regressor

(A) Naive approach

n_estimators	Training data set MAE	Training data set RMSE	Test data set MAE	Test data set RMSE
20	0.083	0.120	0.202	0.317
40	0.052	0.074	0.204	0.316
60	0.034	0.048	0.206	0.316
80	0.023	0.032	0.205	0.315
100	0.014	0.020	0.205	0.314
120	0.009	0.013	0.204	0.313
140	0.006	0.009	0.204	0.313
160	0.004	0.006	0.204	0.313
180	0.003	0.004	0.204	0.313
200	0.002	0.003	0.204	0.313
220	0.001	0.002	0.204	0.313
240	0.001	0.001	0.204	0.313
260	0.001	0.001	0.204	0.313

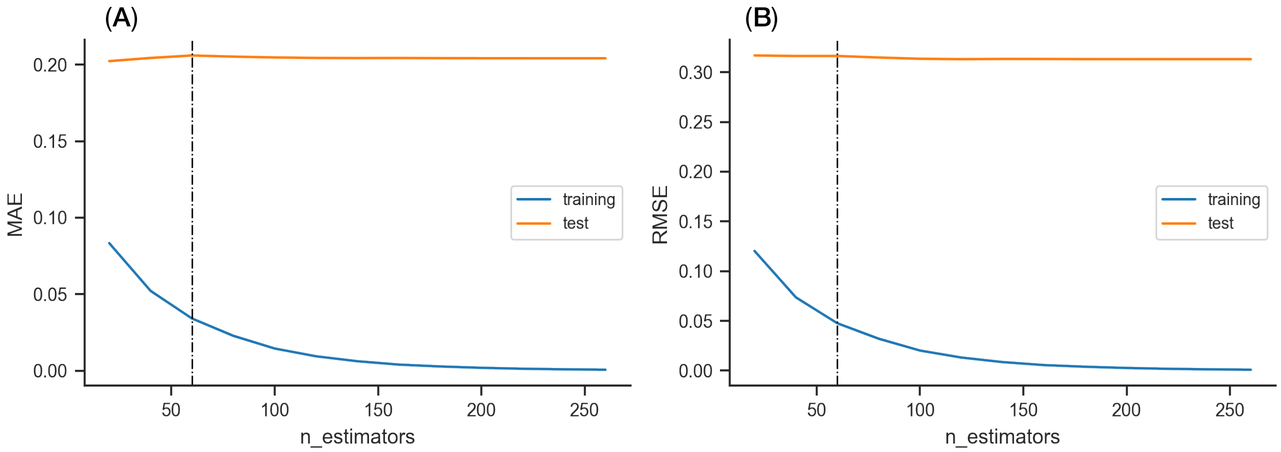

max_depth	Training data set MAE	Training data set RMSE	Test data set MAE	Test data set RMSE
1	0.327	0.438	0.394	0.542
2	0.193	0.256	0.280	0.389
3	0.121	0.162	0.242	0.347
4	0.067	0.092	0.216	0.323
5	0.036	0.049	0.213	0.321
6	0.014	0.020	0.205	0.314
7	0.006	0.008	0.192	0.299
8	0.002	0.003	0.189	0.306
9	0.001	0.001	0.204	0.324

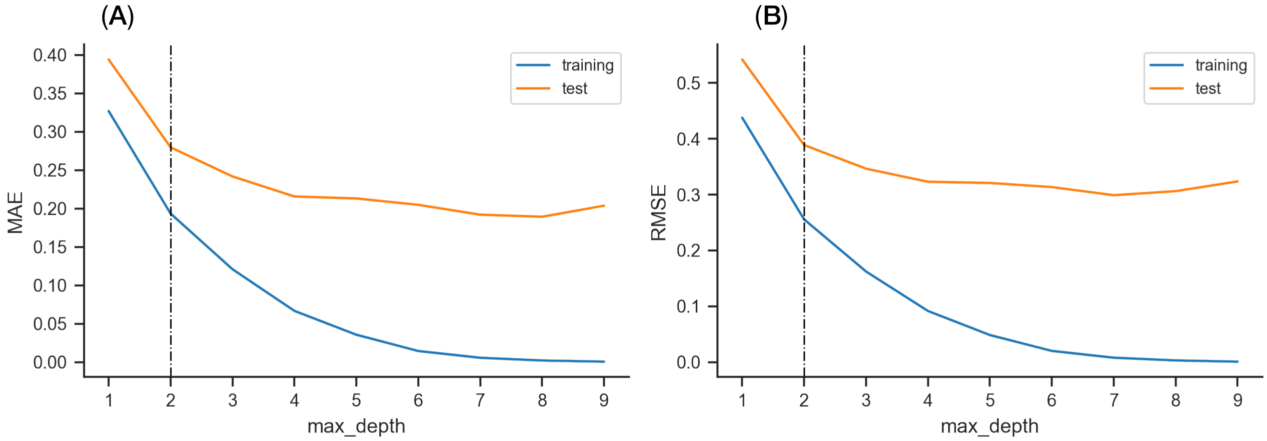

learning_rate	Training data set		Test data set	
	MAE	RMSE	MAE	RMSE
0.00	7.250	7.521	7.101	7.394
0.05	0.094	0.124	0.195	0.285
0.10	0.053	0.076	0.176	0.270
0.15	0.038	0.054	0.185	0.287
0.20	0.028	0.039	0.190	0.290

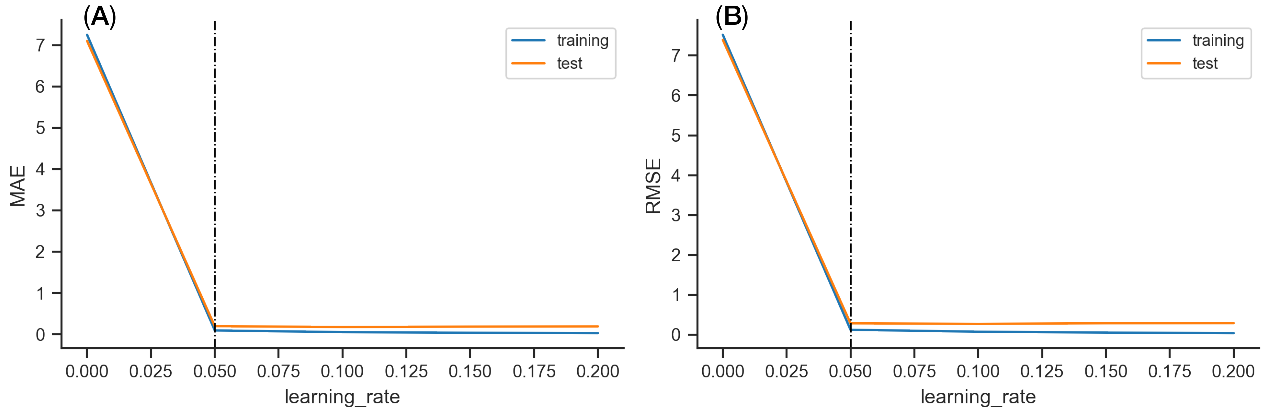

min_child_weight	Training data set		Test data set	
	MAE	RMSE	MAE	RMSE
0	0.014	0.020	0.205	0.314
1	0.014	0.020	0.205	0.314
2	0.014	0.020	0.196	0.302
3	0.014	0.019	0.201	0.307
4	0.015	0.021	0.207	0.320
5	0.015	0.021	0.202	0.307
6	0.018	0.026	0.212	0.318
7	0.017	0.025	0.205	0.313

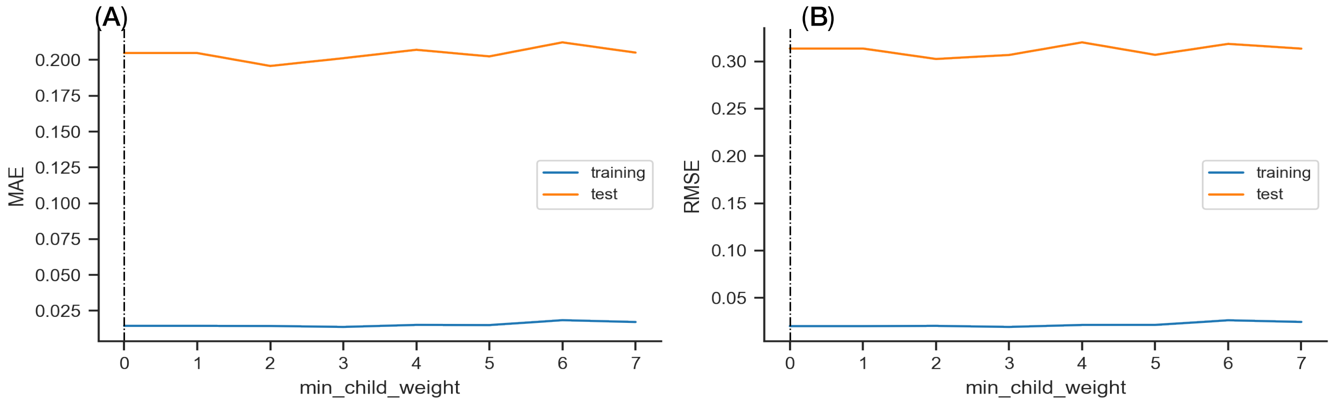

(B) Comparison naive approach vs RandomGridSearch

	n_estimators	max_depth	learning_rate	min_child_weight
**naive approach**	60	2	0.05	0
**(Random)GirdSearch**	250	9	0.05	3

performance of XGBoost Regressor for pH data	Training data set		Test data set	
	MAE	RMSE	MAE	RMSE
**naive approach**	0.524	0.615	0.562	0.669
**(Random)GirdSearch**	0.008	0.011	0.17	0.271

Hyperparameter optimization for the O2 data set using a Random Forest Regressor

(A) Naive approach

n_estimators	Training data set		Test data set	
	MAE	RMSE	MAE	RMSE
1	0.734	5.628	1.369	7.669
5	0.658	3.155	1.583	6.415
10	0.625	2.532	1.687	6.160
15	0.549	2.173	1.347	5.398
20	0.576	2.203	1.572	5.884
30	0.575	2.167	1.413	5.347
40	0.580	2.046	1.380	5.235
50	0.542	1.978	1.417	5.070
60	0.572	2.005	1.395	5.129
70	0.573	1.959	1.418	5.242
80	0.561	1.963	1.416	5.237
90	0.560	2.008	1.428	5.308
100	0.590	2.017	1.405	5.148
150	0.563	1.885	1.408	5.084
200	0.563	1.887	1.403	4.983
250	0.577	1.947	1.460	5.224

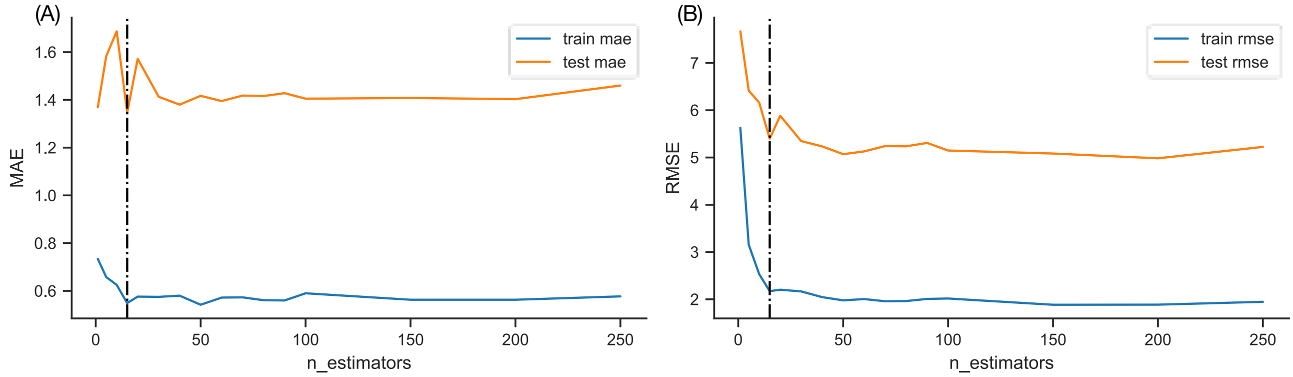

min_weight_fraction_leaf	Training data set		Test data set	
	mae	rmse	mae	rmse
0.00	0.569	2.180	1.447	5.694
0.05	5.430	10.230	5.562	10.572
0.10	9.394	15.004	9.654	15.532
0.15	11.265	17.071	11.465	17.488
0.20	11.034	17.806	11.047	18.433
0.25	17.180	27.107	17.888	28.413
0.30	17.199	27.369	18.111	28.769
0.35	17.288	27.513	18.324	29.129
0.40	17.152	27.292	18.119	28.769
0.45	17.055	28.653	17.492	29.263

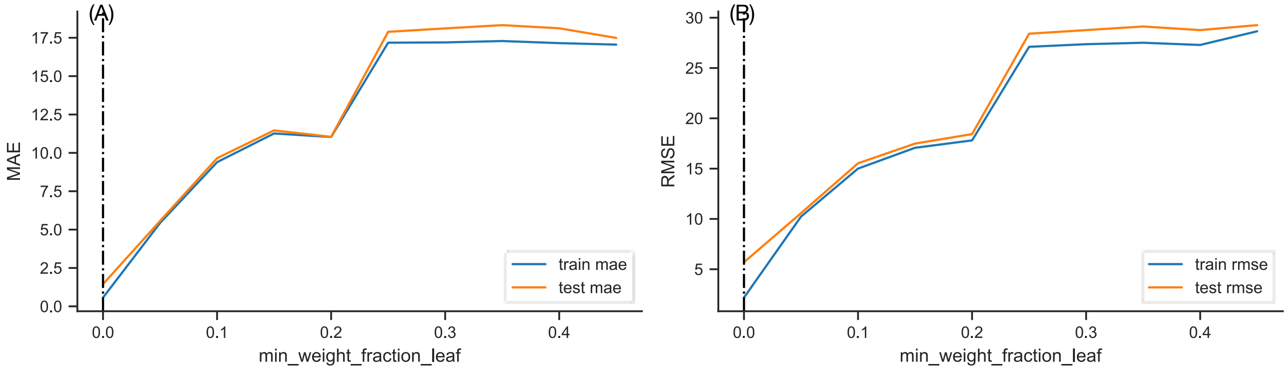

min_samples_split	Training data set		Test data set	
	mae	rmse	mae	rmse
0.5	17.140	27.307	18.033	28.661
2	0.569	2.247	1.470	5.385
3	0.577	2.266	1.589	5.542
4	0.613	2.363	1.279	4.582
5	0.590	2.381	1.444	5.806
6	0.663	2.495	1.464	5.188
7	0.668	2.471	1.378	4.874
8	0.675	2.552	1.483	5.563
9	0.737	2.677	1.531	5.521
10	0.704	2.556	1.413	5.157
20	0.737	2.555	1.328	4.976
50	0.956	3.034	1.614	5.486

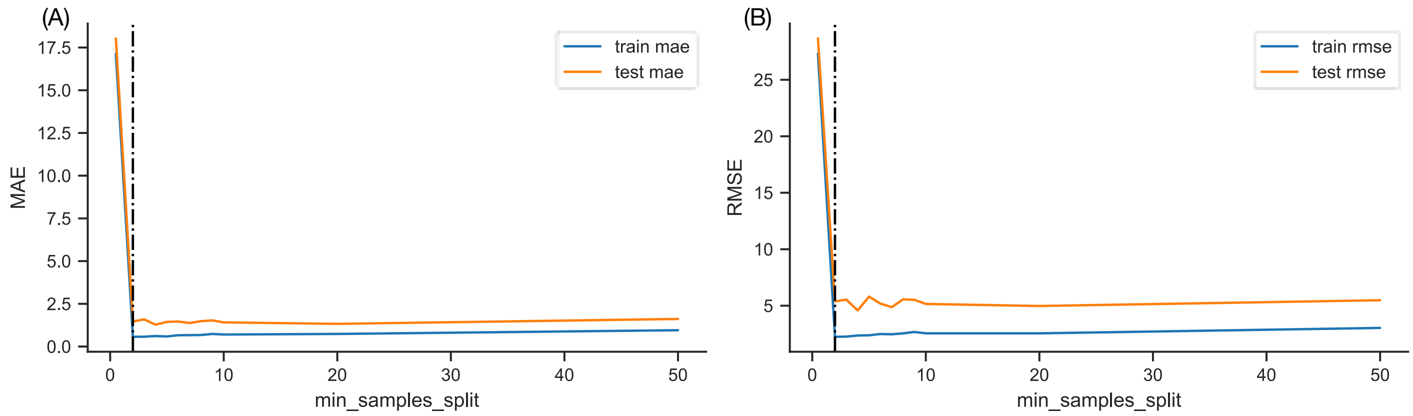

min_samples_leaf	Training data set		Test data set	
	mae	rmse	mae	rmse
1	0.612	2.378	1.239	4.732
2	0.707	2.639	1.411	5.200
3	0.828	2.864	1.330	4.964
4	0.838	3.032	1.366	5.156
5	0.960	3.246	1.383	4.907
6	1.018	3.315	1.300	4.222
7	1.018	3.344	1.406	4.824
8	1.083	3.553	1.519	5.355
9	1.135	3.677	1.437	4.979
10	1.140	3.674	1.478	5.093
20	1.526	4.388	1.937	6.153
50	1.853	4.991	2.057	5.871

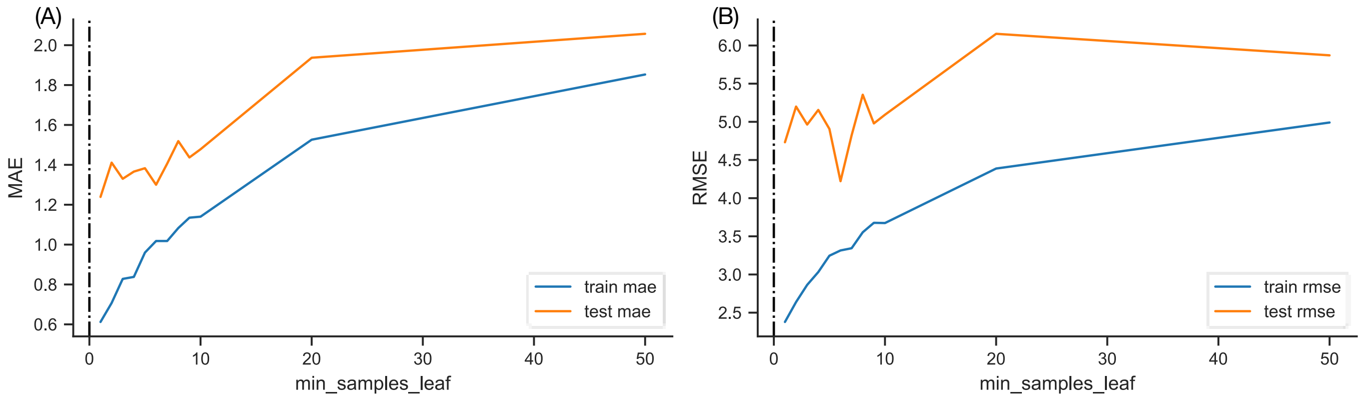

max_leaf_nodes	Training data set		Test data set	
	mae	rmse	mae	rmse
2	17.144	27.289	17.994	28.598
5	6.804	12.616	6.136	11.956
10	2.693	5.303	2.888	6.363
20	1.783	3.693	2.233	5.880
30	1.337	2.870	1.894	5.710
40	1.033	2.440	1.768	5.447
50	0.785	2.500	1.730	6.069
60	0.568	2.150	1.341	4.913
70	0.603	2.261	1.362	4.602
75	0.569	2.259	1.497	5.840
80	0.566	2.159	1.567	5.999
90	0.548	2.262	1.313	5.163
100	0.595	2.387	1.342	5.460
150	0.566	2.308	1.227	4.770
200	0.551	2.260	1.372	5.316

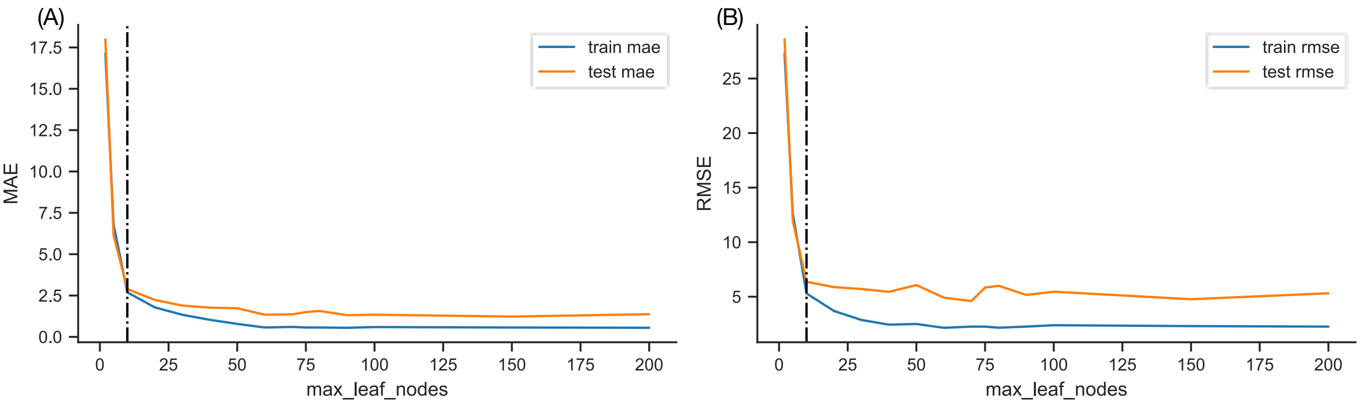

max_features	Training data set		Test data set	
	mae	rmse	mae	rmse
0.5	1.733	3.577	1.954	4.830
1	7.066	10.303	6.890	10.099
2	4.339	7.460	4.301	7.553
3	3.298	5.859	2.907	5.675
4	2.808	5.138	2.731	5.149
5	2.693	5.061	2.502	4.834
6	2.504	4.616	2.329	4.563
7	2.340	4.529	2.151	4.268
8	2.421	4.578	2.295	4.593
9	2.224	4.248	2.172	4.667
10	2.284	4.274	2.204	4.321
15	1.999	3.918	2.003	3.967
20	1.879	3.671	1.826	3.726
30	1.964	3.795	1.759	3.389

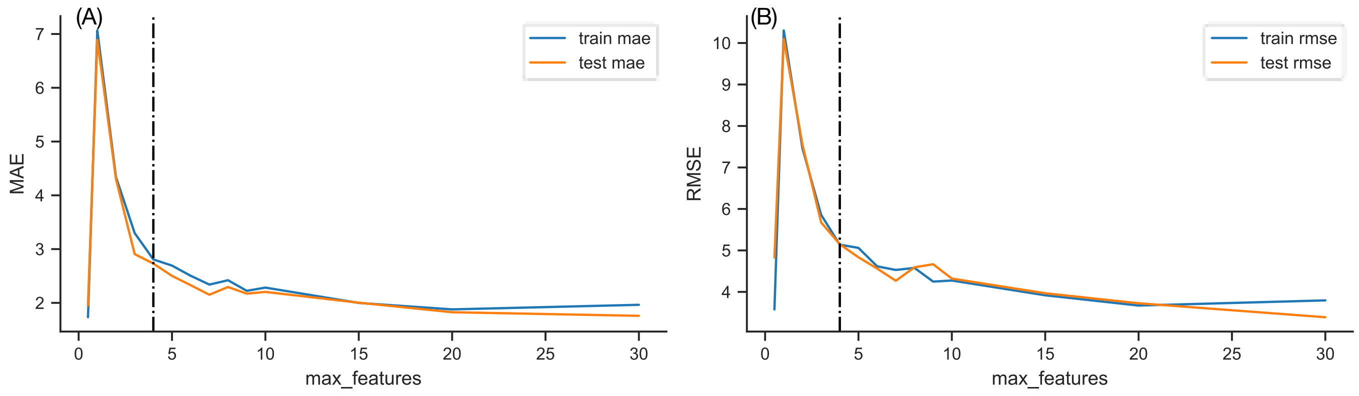

max_depth	Training data set mae	Training data set rmse	Test data set mae	Test data set rmse
1	22.028	29.044	23.363	31.576
2	10.799	15.690	10.412	15.593
3	6.226	10.266	5.557	9.402
4	3.993	6.784	3.789	6.792
5	3.196	5.780	2.761	4.860
6	3.407	5.955	3.155	5.469
7	2.855	5.324	2.662	5.155
8	2.888	5.260	2.757	5.386
9	2.849	5.189	2.643	4.593
10	3.134	5.747	2.891	5.921
15	2.887	5.301	2.758	5.416
20	2.784	5.330	2.604	5.314
30	2.893	5.223	2.789	5.085
40	2.997	5.396	2.838	5.310
50	2.851	5.278	2.670	5.068
60	2.938	5.454	2.818	5.559
70	2.913	5.240	2.655	5.119
80	2.927	5.428	2.827	5.750
90	2.996	5.489	2.724	5.215
100	2.991	5.592	2.713	5.337
200	2.921	5.175	2.756	5.385

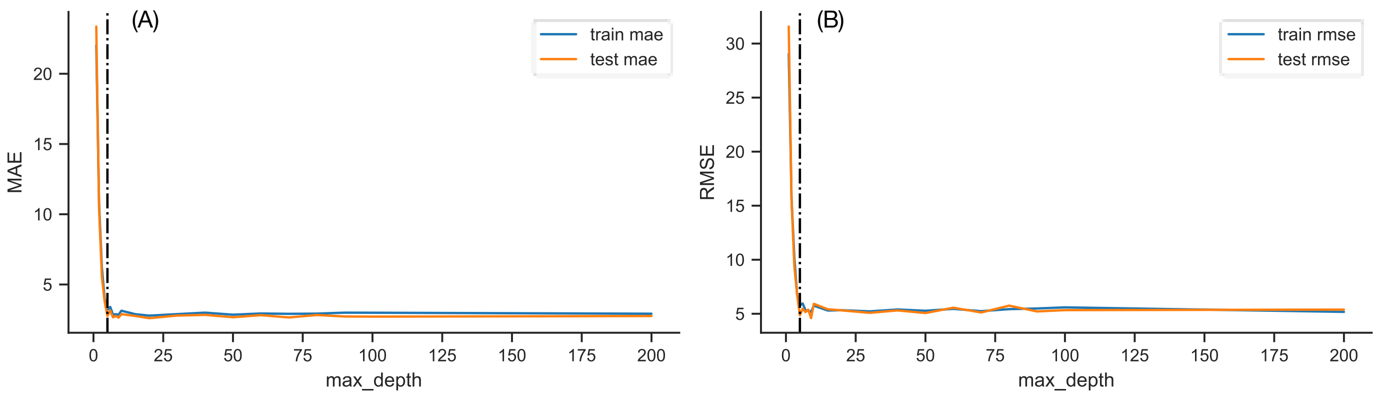

bootstrap	Training data set		Test data set	
	mae	rmse	mae	rmse
FALSE	3.153	5.651	2.862	5.537
TRUE	3.114	5.754	2.763	5.240

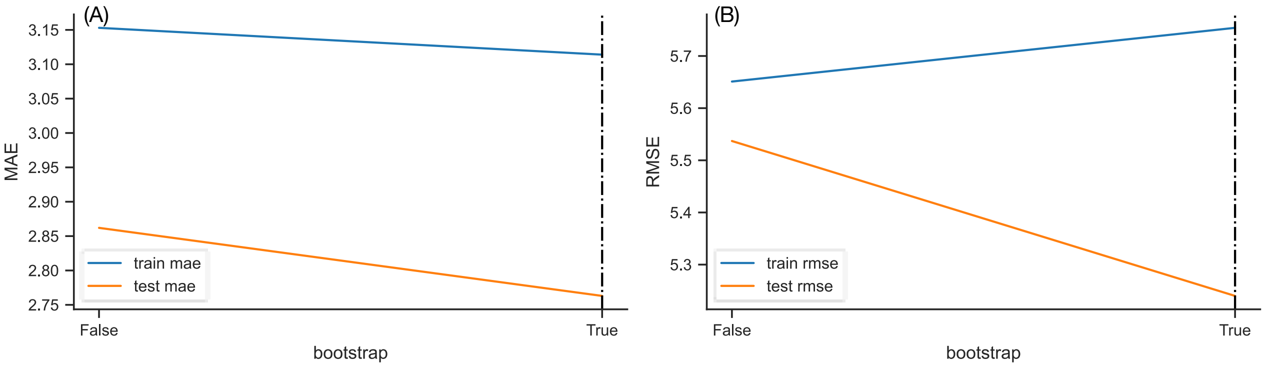

(B) Comparison naive approach vs RandomGridSearch

	naive approach	om)GirdSearch
n_estimators	15	100
min_weight_fra	0	0
min_samples_s	2	3
min_samples_l	1	4
max_leaf_node	20	90
max_features	4	10
max_depth	5	30
bootstrap	TRUE	FALSE

performance of Random Forest Regressor for O2 data	Training data set		Test data set	
	MAE	RMSE	MAE	RMSE
**naive approach**	3.120	5.485	2.888	5.388
**(Random)GirdSearch**	0.738	1.934	1.439	3.718

Hyperparameter optimization for the O2 data set using a Decision Tree Regressor

(A) Naive approach

splitter	Training data set MAE	Training data set RMSE	Test data set MAE	Test data set RMSE
best	1.31E-13	2.11E-13	0.938	6.519
random	1.01E-13	1.84E-13	0.569	5.038

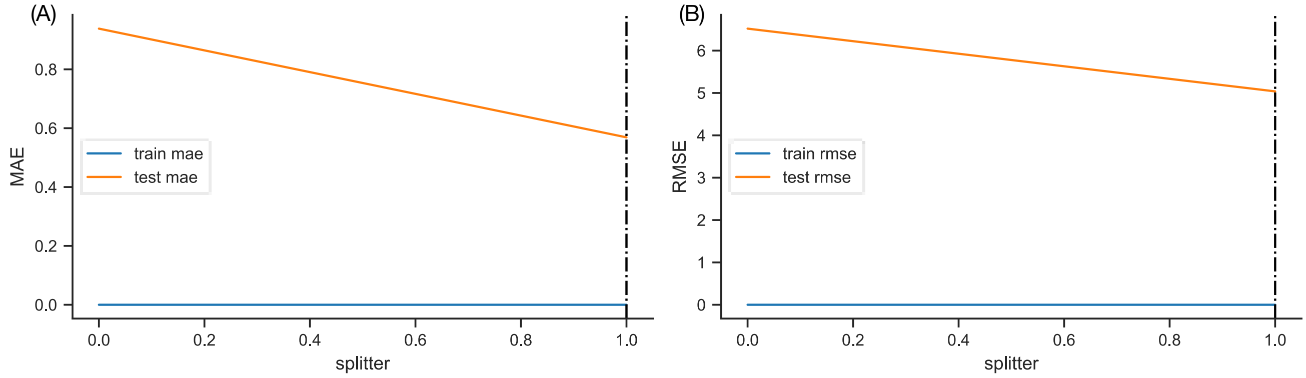

max_depth	Training data set mae	Training data set rmse	Test data set mae	Test data set rmse
1	18.161	27.920	18.760	28.787
2	11.515	18.293	10.943	17.463
3	7.391	12.257	6.593	11.688
4	4.020	8.903	4.097	9.650
5	3.650	8.686	3.670	9.388
6	3.535	9.464	2.728	7.582
7	3.448	8.929	2.622	6.293
8	5.230	10.876	4.890	10.128
9	4.221	9.553	3.826	9.604
10	4.592	9.260	3.875	7.785
15	4.829	10.098	5.061	10.962
20	4.727	9.264	4.216	8.479
25	4.587	11.033	4.216	10.460
30	5.098	10.742	4.400	9.072
50	4.666	10.454	4.104	9.337

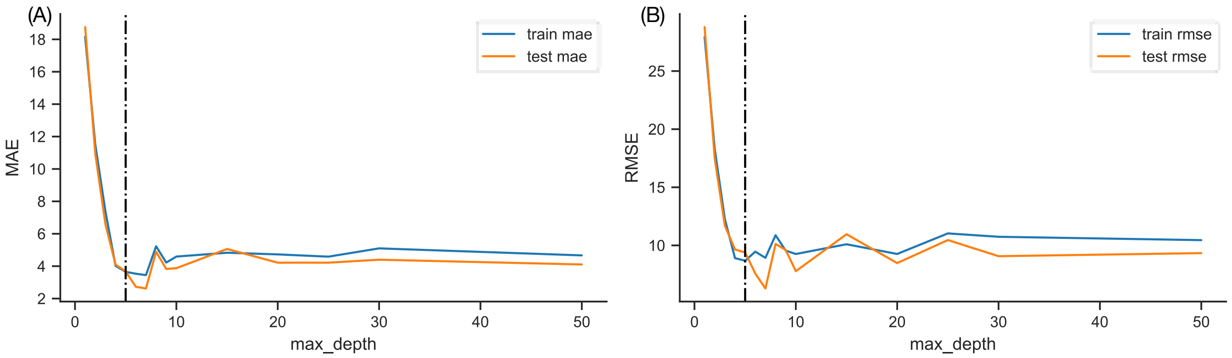

	Training data set		Test data set	
min_samples_leaf	mae	rmse	mae	rmse
0.1	11.182	16.562	11.437	16.718
0.2	18.431	27.728	19.279	28.728
0.25	19.551	28.110	19.827	28.198
0.5	51.139	57.905	53.864	61.216
1	0.000	0.000	0.957	6.719
2	0.155	1.825	1.071	6.934
3	0.438	3.200	1.313	7.175
4	0.494	3.222	0.592	4.415
5	0.804	4.080	0.989	4.969
6	0.752	3.977	0.798	4.435
7	0.986	4.682	0.922	4.601
8	1.035	4.652	1.124	4.600
9	1.098	4.997	1.370	6.121
10	1.315	5.231	1.170	5.074
15	1.419	5.429	1.377	6.348
20	1.679	5.825	1.656	5.275

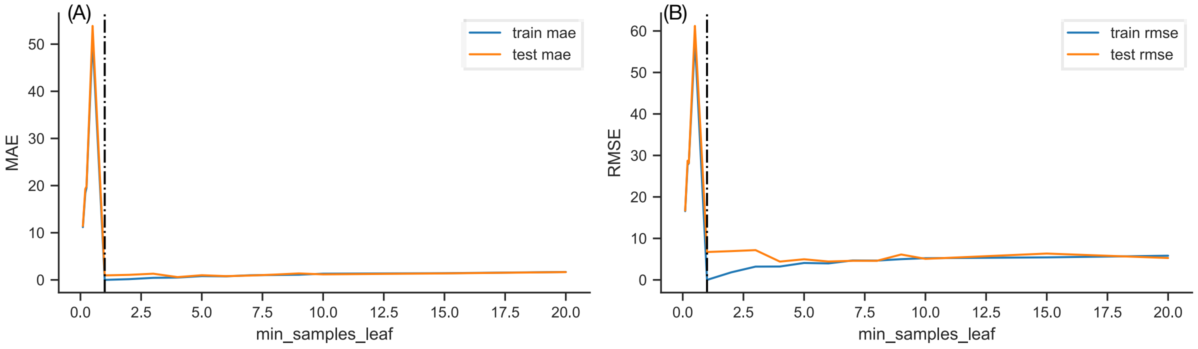

	Training data set		Test data set	
min_weight_fraction_leaf	mae	rmse	mae	rmse
0	0.000	0.000	1.390	8.369
0.05	7.640	13.207	7.145	12.161
0.1	12.407	18.288	12.513	18.237
0.15	12.219	18.531	11.746	17.678
0.2	12.342	20.495	12.174	20.660
0.25	18.479	27.756	19.084	28.811
0.3	20.869	28.870	20.316	28.385
0.35	17.430	28.845	17.744	29.147
0.4	18.967	28.294	19.662	29.350
0.45	19.320	34.414	18.920	33.802

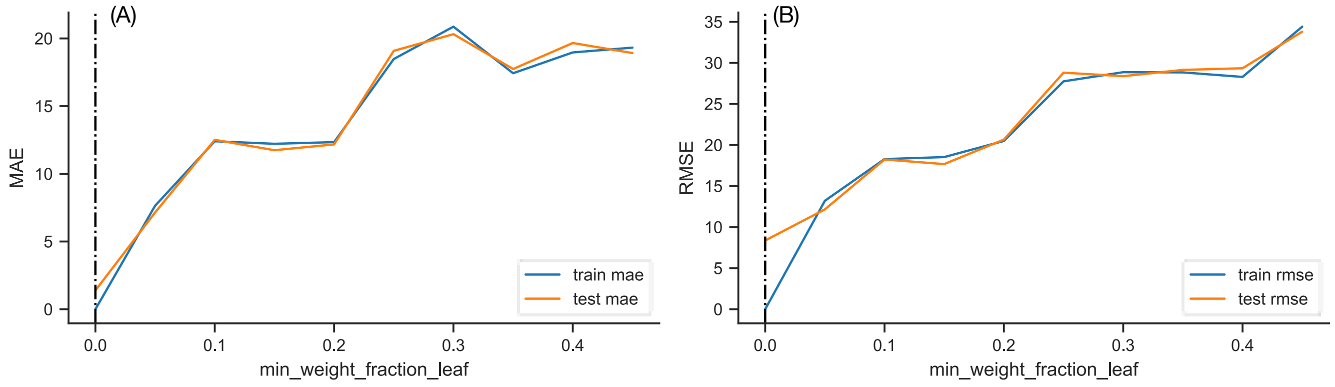

max_features	Training data set mae	Training data set rmse	Test data set mae	Test data set rmse
None	3.353	9.060	3.212	9.334
0.5	4.259	10.107	4.357	11.300
1	20.764	26.470	20.129	25.459
2	27.083	37.421	29.430	41.126
3	16.135	27.760	16.583	28.829
4	12.947	19.107	13.516	20.864
5	10.421	18.768	9.152	16.509
6	6.879	12.921	6.433	12.531
7	9.988	16.788	8.839	15.685
8	8.219	14.647	7.006	12.575
9	9.296	14.557	9.349	14.280
10	7.627	13.865	6.319	11.741
15	10.237	15.652	9.647	14.609
20	5.946	11.899	5.128	9.978
50	4.448	10.160	3.546	7.698

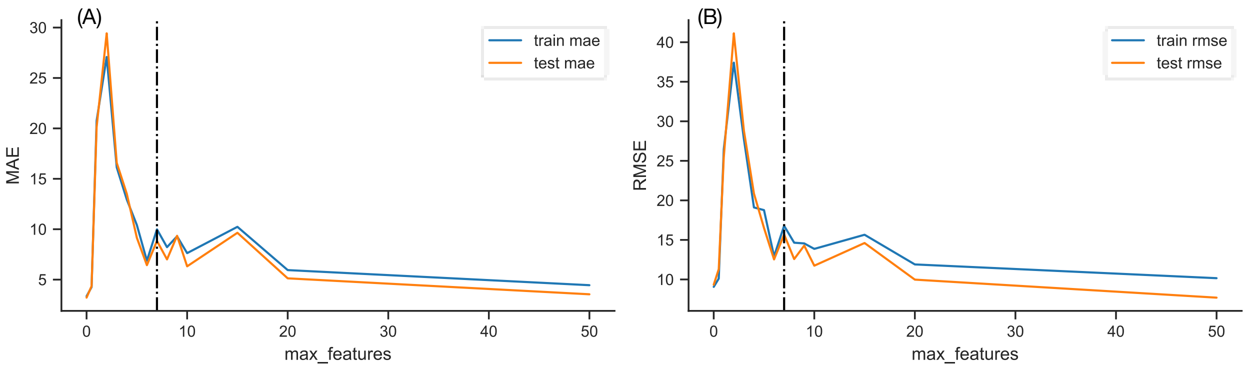

max_leaf_nodes	Training data set mae	Training data set rmse	Test data set mae	Test data set rmse
2	24.679	31.254	24.663	31.341
3	13.927	20.620	13.424	19.537
4	12.198	18.278	12.637	18.750
5	7.688	14.985	6.416	13.391
6	7.712	13.868	6.481	12.050
7	6.222	11.592	5.924	11.140
8	6.328	12.428	5.679	11.873
9	4.175	9.794	3.455	8.376
10	3.801	9.884	2.734	7.374
15	3.204	7.871	2.607	6.356
20	2.495	6.012	1.976	4.433
50	1.036	3.138	1.540	6.330
75	0.429	0.965	1.236	6.034
80	0.340	0.758	1.056	6.034
90	0.370	0.759	1.399	7.353
100	0.331	0.817	1.215	6.863
110	0.206	0.515	0.902	5.899
120	0.140	0.374	1.463	8.163
130	0.003	0.019	0.812	5.771
140	0.000	0.001	0.311	2.962
150	0.066	0.152	1.665	8.745

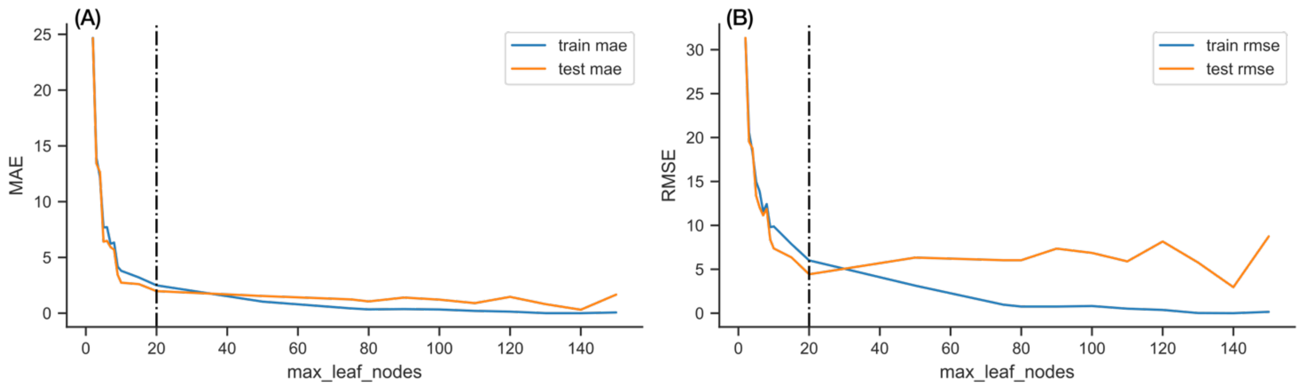

(B) Comparison naive approach vs RandomGridSearch

	splitter	max_features	max_depth	min_weight_fra	min_samples_leaf	max_leaf_nodes
**naive approach**	random	7	5	0	1	20
**(Random)GirdSearch**	best	7	10	0	3	100

performance of Decision Tree Regressor for O2 data	Training data set		Test data set	
	MAE	RMSE	MAE	RMSE
**naive approach**	4.835	10.276	4.948	10.466
**(Random)GirdSearch**	0.536	2.681	0.997	4.943

Hyperparameter optimization for the O2 data set using a XGBoost Regressor

(A) Naive approach

n_estimators	Training data set		Test data set	
	MAE	RMSE	MAE	RMSE
0	143.648	154.842	138.947	151.713
5	24.318	26.388	23.404	25.909
10	4.243	4.871	4.760	6.645
20	0.563	0.988	1.625	5.008
30	0.421	0.723	1.591	4.997
40	0.328	0.555	1.568	4.993
50	0.255	0.425	1.551	4.990
100	0.110	0.165	1.541	4.982
150	0.048	0.068	1.540	4.985
200	0.022	0.031	1.540	4.986
250	0.010	0.014	1.541	4.986
300	0.004	0.006	1.541	4.985

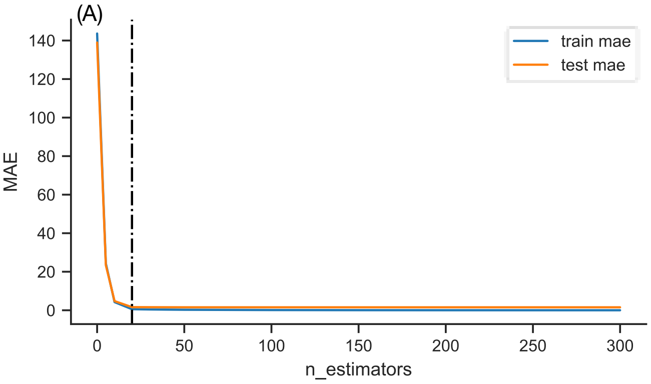

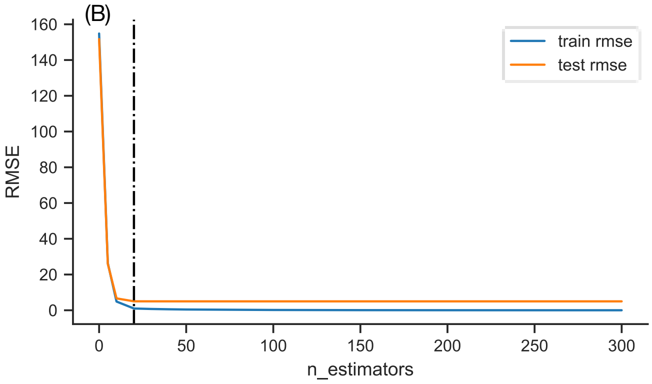

max_depth	Training data set		Test data set	
	MAE	RMSE	MAE	RMSE
0	0.563	0.988	1.625	5.008
1	5.705	9.464	5.395	9.603
2	4.128	6.807	4.235	7.726
3	2.154	3.864	2.598	5.204
4	1.346	2.605	2.191	5.465
5	0.858	1.669	1.762	5.012
6	0.563	0.988	1.625	5.008
7	0.416	0.748	1.512	5.119
8	0.321	0.559	1.602	5.303
9	0.294	0.522	1.523	5.199
10	0.250	0.455	1.412	5.097

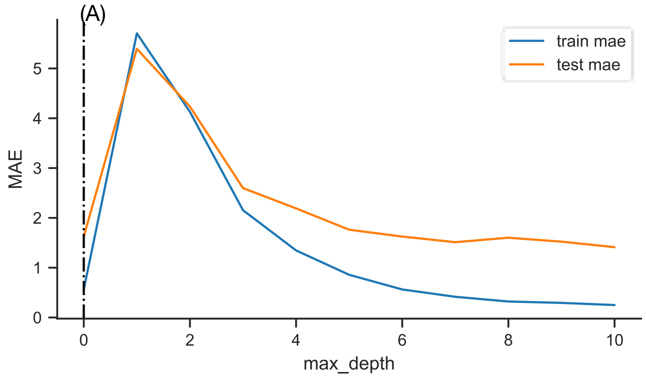

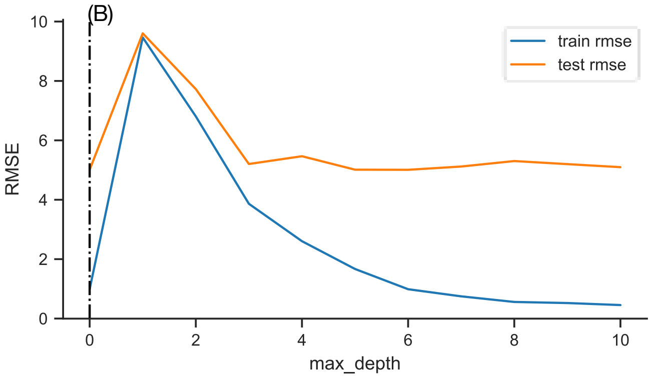

learning_rate	Training data set		Test data set	
	MAE	RMSE	MAE	RMSE
0	143.648	154.842	138.947	151.713
0.05	51.654	55.789	49.975	54.744
0.1	17.600	19.190	17.130	19.121
0.15	5.783	6.502	6.076	7.760
0.2	1.981	2.642	2.641	5.296
0.25	0.993	1.761	1.908	5.281
0.3	0.858	1.669	1.762	5.012
0.35	0.859	1.543	2.082	5.489
0.4	0.834	1.474	1.950	5.362
0.45	0.773	1.379	1.994	5.215
0.5	0.831	1.420	2.181	5.507
1	0.657	1.020	3.382	9.751

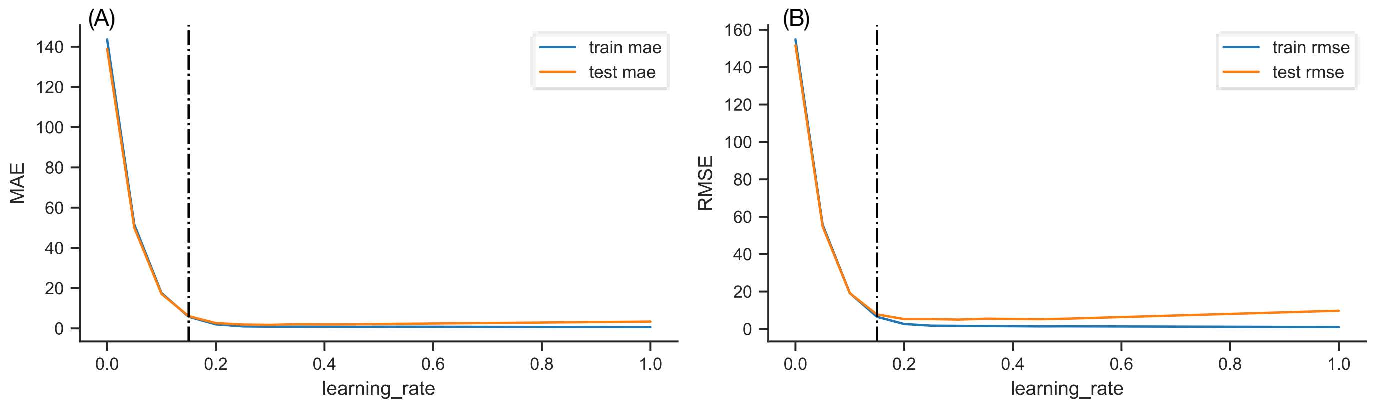

min_child_weight	Training data set		Test data set	
	MAE	RMSE	MAE	RMSE
0	5.783	6.502	6.076	7.760
1	5.783	6.502	6.076	7.760
2	5.837	6.553	6.034	7.549
3	5.826	6.557	6.072	7.611
4	5.876	6.610	6.026	7.392
5	5.867	6.610	6.021	7.413
6	5.873	6.632	6.004	7.333
7	5.883	6.622	6.070	7.463
8	5.889	6.645	5.994	7.434
9	5.900	6.656	6.035	7.478
10	5.897	6.655	6.026	7.435
15	5.919	6.703	6.037	7.434
20	5.957	6.758	6.144	7.577
30	5.988	6.835	6.170	7.716
40	6.012	6.886	6.159	7.750
50	6.072	6.945	6.113	7.702

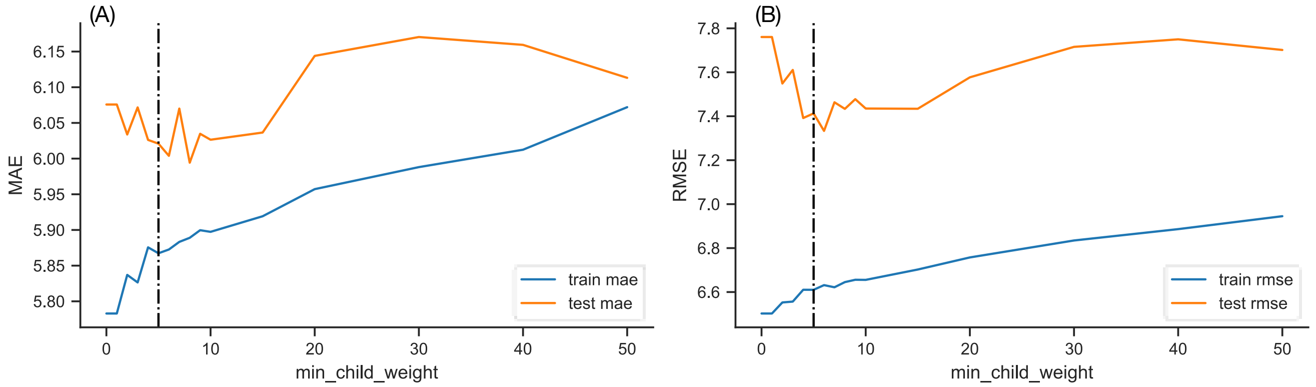

(B) Comparison naive approach vs RandomGridSearch

	n_estimators	max_depth	learning_rate	min_child_weight
**naive approach**	20	0	0.15	5
**(Random)GirdSearch**	250	5	0.05	7

performance of XGBoost Regressor for O2 data	Training data set		Test data set	
	MAE	RMSE	MAE	RMSE
**naive approach**	5.783	6.502	6.076	7.760
**(Random)GirdSearch**	0.585	1.068	1.668	4.541
